# Distinct time-dependent *S*. Typhi-specific CD8^+^ T cell programs are associated with clinical outcome following Ty21a immunization and wild-type *S*. Typhi challenge in humans

**DOI:** 10.3389/fcimb.2026.1924754

**Published:** 2026-10-02

**Authors:** Jayaum S. Booth, Rekha R. Rapaka, Monica A. McArthur, Stephanie Fresnay, Thomas C. Darton, Bing Guo, Claire S. Waddington, Myron M. Levine, Andrew J. Pollard, Marcelo B. Sztein

**Affiliations:** 1 Center for Vaccine Development and Global Health, University of Maryland School of Medicine, Baltimore, MD, United States; 2 Department of Pediatrics, University of Maryland School of Medicine, Baltimore, MD, United States; 3 Department of Medicine, University of Maryland School of Medicine, Baltimore, MD, United States; 4 Global Clinical Development, Sanofi, Swiftwater, PA, United States; 5 GSK, Cambridge, MA, United States; 6 Oxford Vaccine Group, Department of Pediatrics, University of Oxford, and the NIHR Oxford Biomedical Research Centre, Oxford, United Kingdom; 7 Clinical Infection Research Group, Division of Clinical Medicine, School of Medicine and Population Health, University of Sheffield, and the NIHR Sheffield Biomedical Research Centre, Sheffield, United Kingdom; 8 Department of Clinical Sciences, Liverpool School of Tropical Medicine, Liverpool, United Kingdom; 9 Tumor Immunology and Immunotherapy Program. University of Maryland Marlene and Stewart Greenebaum Comprehensive Cancer Center, Baltimore, MD, United States

**Keywords:** CD8^+^ T cell response, controlled human infection model (CHIM), high dimensional profiling, *S. Typhi* infection, Ty21a immunization, typhoid

## Abstract

**Introduction:**

CD8^+^ T cells are essential components of the adaptive immune system that play a critical role in determining the clinical severity of typhoid disease by directly killing *Salmonella enterica* serovar Typhi (*S*. Typhi)-infected cells, producing cytokines, chemokines, and establishing memory responses that have been linked to clinical outcome in a controlled human infection model (CHIM) of typhoid. Despite decades of extensive research, the role of memory CD8^+^ T (T_M_) cells and its subsets as correlates of protection against *S*. Typhi infection and typhoid disease remains incompletely understood.

**Methods:**

In this study, we used specimens from a typhoid CHIM study in which participants who were immunized with live attenuated *S*. Typhi vaccine, Ty21a, were subsequently challenged with virulent *S*. Typhi. Some participants developed typhoid disease (TD) and some did not (NoTD), allowing us to assess the immune program of CD8^+^ T_M_ subsets involved in the prevention of typhoid disease using mass cytometry, CyTOF.

**Results and discussion:**

Our high-dimensional profiling data showed that TD and NoTD participants followed divergent memory CD8^+^ T cell trajectories. We observed that CD8^+^ T clusters from NoTD participants are enriched for early multifunctional, protection-associated programs while TD participants exhibited a distinct clustering associated with susceptibility states. Specifically, using unsupervised dimensionality reduction and clustering analysis, T-REX, and feature-ranking analyses, we identified memory CD8^+^ T cell subsets including effector/memory (T_EM_), T central/memory (T_CM_) and CD45RA+ T_EM_ (T_EMRA_) associated with clinical outcomes. CD8^+^ T_EM_ from NoTD participants were generally characterized by stronger effector-associated signatures such as CXCR3, IL-17A, TNFα, and MIP1b. In contrast, CD8^+^ T_EM_ from TD participants showed CCR6- and integrin α4β7-biased immune profiles. Detailed analysis revealed that CD8⁺ T_CM_ and T_EMRA_ subsets also contained distinct outcome-associated states. These patterns were confirmed by conventional supervised gating that validated both protection-associated and susceptibility-associated marker combinations across CD8^+^ T_EM_, T_CM_ and T_EMRA_ subsets. These data suggest that: 1. Protection and susceptibility are not restricted to one CD8^+^ T cell subset but rather are distributed across the broader CD8^+^ T memory landscape and, 2. Typhoid clinical outcome is associated with qualitatively distinct CD8^+^ T memory programs rather than differences only in the magnitude of the overall anti-*S*. Typhi immune response.

## 1 Introduction

The human-restricted bacterial pathogen *Salmonella enterica serovar* Typhi (*S*. Typhi) causes typhoid fever persists as a global health challenge in many low and middle-income countries and poses a risk for travelers from high-income countries. The global typhoid burden is exacerbated by the emergence and spread of antimicrobial-resistant *Salmonella* strains (Klemm et al., 2018; Jamilah et al., 2020). Although licensed vaccines, including Vi conjugate typhoid vaccines and Ty21a live oral vaccine, have shown efficacy in endemic settings (Levine et al., 1987; Simanjuntak et al., 1991; Levine et al., 1999; Shakya et al., 2019; Patel et al., 2021; Qadri et al., 2021; Shakya et al., 2021; Neuzil et al., 2024), vaccine performance varies somewhat across formulations, populations and settings, and the immune mechanisms that mediate protection against typhoid disease in humans remain incompletely defined (Batool et al., 2024). The continued absence of validated immunological correlates of protection remains a major barrier to efficient attenuated vaccine down-selection, iterative vaccine optimization, and the use of immunobridging strategies in clinical development. Controlled human infection models (CHIMs) that assess typhoid vaccine efficacy provide a practical approach to address these gaps by providing insight into host-pathogen interactions with well-defined exposure and prospective clinical endpoints and intensive longitudinal sampling, enabling direct interrogation of immune features that precede and potentially determine clinical outcomes (Sztein and Booth, 2022). Accordingly, in this study, we used peripheral blood mononuclear cells (PBMC) samples from an Oxford typhoid CHIM (Waddington et al., 2014) to identify immune programs associated with protection versus clinical disease progression.

The induction of humoral and cell mediated immune responses (CMI) have been widely studied in PBMC obtained from healthy volunteers following immunization with 4 doses of Ty21a (Kantele, 1990; Salerno-Goncalves et al., 2002; Salerno-Goncalves et al., 2004; Salerno-Goncalves et al., 2005; Sztein, 2007; Salerno-Goncalves et al., 2010; Lindow et al., 2011; McArthur and Sztein, 2012; Sztein et al., 2014; Sztein, 2017). These studies have shown that live oral *S*. Typhi vaccines induced both CD4^+^ and CD8^+^ T cell responses, including IFNγ, cytotoxic T cells (CTL), proliferation, and multifunctional (MF) antigen-specific cytokine-producing cells (Sztein et al., 1994; Sztein et al., 1995; Salerno-Goncalves et al., 2002; Salerno-Goncalves et al., 2004; Salerno-Goncalves et al., 2005; Sztein, 2007; Salerno-Goncalves et al., 2010; McArthur and Sztein, 2012; Sztein et al., 2014; Wahid et al., 2014; McArthur et al., 2015; Wahid et al., 2015; Fresnay et al., 2016; Wahid et al., 2016; Fresnay et al., 2017; Salerno-Goncalves et al., 2017a; Salerno-Goncalves et al., 2017b; Sztein, 2017), which might play a key role in long-term immunity. Our group has also reported that Ty21a elicits *S*. Typhi-specific CD4^+^ and CD8^+^ T cell responses in PBMC by various T memory (T_M_) cell subsets, including T central/memory (T_CM_), T effector/memory (T_EM_), and CD45RA^+^ T_EM_ (T_EMRA_) (Salerno-Goncalves et al., 2010; Wahid et al., 2015). These responses were predominantly in the T_EM_ and T_EMRA_ subsets with low magnitude responses also observed in CD4^+^ and CD8^+^ T_CM_ subsets (Sztein et al., 1995; Sztein et al., 2014; Wahid et al., 2015). Furthermore, *S.* Typhi-specific MF cells were increased in both CD4^+^ and CD8^+^ T_EM_ and T_EMRA_ subsets post-vaccination albeit with differences in response between subsets. However, these detailed CMI responses were not evaluated in a challenge setting and their contribution to protection against disease remained unclear. In the current study we undertook to correlate CD8^+^ CMI responses to clinical protection.

Using PBMC samples obtained from a typhoid CHIM study that was performed at the University of Oxford Vaccine group (OVG) (Waddington et al., 2014), we have previously demonstrated that baseline *S*. Typhi-responsive multifunctional (MF) CD8^+^ T cell immunity is linked to clinical outcome following oral wild type (wt) *S*. Typhi challenge. In a low-dose (1-5 X10^3^ cfu of *S*. Typhi) challenge cohort, higher MF baseline CD8^+^ T responses were associated with protection and delayed time to diagnosis, while typhoid diagnosis coincided with decreases in circulating *S*. Typhi-specific CD8^+^ effector memory T cells (T_EM_) exhibiting gut-homing potential, consistent with trafficking to tissues during infection (Fresnay et al., 2016). In a subsequent high-dose challenge study, we again observed that *S*. Typhi-responsive CD8^+^ memory responses at baseline related to clinical outcome but with a more nuanced pattern, i.e., higher baseline responses were associated with increased risk of meeting diagnostic criteria. However, among participants who developed typhoid, stronger baseline responses were associated with longer time to diagnosis (Fresnay et al., 2017). These findings suggested that pre-existing CD8^+^ T cell immunity may influence both early containment and inflammatory trajectories, and that the qualitative composition of the response, not simply the magnitude of these responses, may be critical, particularly when exposed to higher infectious doses. In parallel, CHIM-based work has underscored that protective immunity to enteric pathogens is likely orchestrated across immune compartments and arms of the adaptive response (McArthur et al., 2015; Toapanta et al., 2015; Toapanta et al., 2016; Salerno-Goncalves et al., 2017a; Booth et al., 2024; Toapanta et al., 2024). For example, circulating T follicular helper (cT_FH_) cells, which provide key help to B cells and shape antibody quality, have emerged as an important component of the human host immune response that integrates cellular and humoral immunity. Building on the typhoid CHIM platform, we recently identified distinct contributions of cT_FH_ subsets across immunization and challenge phases, with patterns consistent with their involvement in the development or prevention of typhoid disease and implications for vaccines aiming to induce durable T cell and antibody responses (Booth et al., 2024). Together, these studies established the typhoid CHIM as a powerful translational tool for the discovery of immunological correlates of protection (CoP) and for defining high-resolution cellular programs associated with protection from disease.

Here, we extend our CHIM studies by applying high-dimensional profiling to map *S*. Typhi-specific CD8^+^ T cell responses across baseline, post-Ty21a immunization, and wild-type (wt) *S*. Typhi challenge, with explicit stratification by typhoid diagnosis. By integrating longitudinal sampling with unbiased discovery and feature-prioritization approaches, we aim to refine CD8^+^ T cell correlates into more precise and translationally actionable signatures that can inform immune monitoring for oral typhoid vaccines and guide next-generation vaccine development.

## 2 Materials and methods

### 2.1 Ethics statement

Participants with no history of typhoid fever or prior typhoid vaccination were enrolled in studies at the University of Oxford and biological samples (e.g., blood) for the wt *S.* Typhi challenge model usage was approved by the National Research Ethics Service (NRES), Oxfordshire Research Ethics Committee A (11/SC/0302) (Darton et al., 2016). The study was conducted in accordance with the 1964 Declaration of Helsinki and the principles of the International Conference on Harmonization Good Clinical Practice guidelines (International Conference on Harmonisation of Technical Requirements for Registration of Pharmaceuticals for Human Use (ICH), 1997). Participants were informed of the purpose and risks of the study, and written informed consent was obtained before enrollment.

### 2.2 Participants and study design

The University of Oxford Professor Pollard’s Oxford Vaccine group recruited and screened ninety-nine participants aged 18–46 years as previously described (Darton et al., 2016). Individuals were excluded if they had previously received typhoid vaccination, and/or had lived for more than 6 months in typhoid-endemic regions, and/or had a prior history of typhoid fever. In one of the arms of the study, participants received three doses of the live oral attenuated typhoid vaccine Ty21a before oral challenge 1–5 × 10^4^ CFU of wild-type *S*. Typhi (Quailes strain), after gastric acid neutralization with NaHCO3 (Darton et al., 2016). Following challenge, participants were classified as having typhoid disease (TD) if they developed blood culture-confirmed *S.* Typhi bacteremia or fever ≥38 °C for at least 12 hours; those who did not meet these criteria were classified as NoTD (Darton et al., 2016). Of the thirty participants who received Ty21a followed by challenge with wild type *S*. Typhi, 13/30 (43.3%) met the prespecified criteria for typhoid diagnosis (TD), whereas 17/30 (56.7%) did not develop typhoid disease (NoTD). Participants were eligible for inclusion in the study if sufficient numbers of viable PBMC specimens were available at the required study time points and met the established sample-quality criteria. Among the eligible participants, eight TD and eight NoTD participants were randomly selected for analysis, providing balanced representation of the two clinical-outcome groups.

Peripheral blood mononuclear cells (PBMC) were collected at multiple time points (**Supplementary Figure 1**), isolated by density-gradient centrifugation, and cryopreserved in liquid nitrogen using standard procedures (Booth et al., 2024). PBMC collected before Ty21a vaccination, pre and post challenge and up to 28 days after challenge were processed within 4 hours of collection and were included in the analyses presented here (**Supplementary Figure 1**).

### 2.3 Generation of autologous target cells and *S*. Typhi infection

Autologous Epstein–Barr virus (EBV)-transformed lymphoblastoid cell lines (B-EBV cells) were generated from PBMC obtained from each participant, as previously described (Sztein et al., 1995; Salerno-Goncalves et al., 2004). Briefly, PBMCs were infected with EBV-containing supernatant from the B95-8 cell line (ATCC CRL-1612) and cultured in the presence of cyclosporine (0.5 μg/ml; Sigma-Aldrich, St. Louis, MO, USA) for 15–30 days to generate B-EBV cells. Autologous B-EBV cells were then infected with wt *S.* Typhi strain ISP1820 at a multiplicity of infection (MOI) of 7:1 (bacteria:target cell ratio) for 3 hours at 37 °C in antibiotic-free Roswell Park Memorial Institute (RPMI) media. Following infection, cells were washed extensively with cRPMI and cultured overnight in cRPMI containing gentamicin (150 μg/ml). Infection efficiency was confirmed by flow cytometry after staining with anti-*Salmonella* common structural antigen (Kierkegaard & Perry, Gaithersburg, MD, USA), as previously described (Salerno-Goncalves et al., 2002).

### 2.4 Stimulation of PBMC and staining for mass cytometry analysis

As previously described, PBMC were thawed and rested overnight in cRPMI prior to stimulation with *S.* Typhi-infected target cells (Fresnay et al., 2016; Fresnay et al., 2017; Booth et al., 2024). Uninfected target cells and Staphylococcal enterotoxin B (SEB; 10 μg/ml) served as negative and positive controls, respectively. Target cells were γ-irradiated (6,000 rad) and co-cultured with PBMC at an effector:stimulator ratio of 7:1 for 2 hours in the presence of metal-conjugated anti-CD107a monoclonal antibody (Standard BioTools). Golgi Stop (0.5 μl; monensin, BD) and Golgi Plug (0.5 μl; brefeldin A, BD) were then added, and cultures were incubated overnight at 37 °C in 5% CO2.

Following stimulation, PBMC were processed for mass cytometry as previously described (Booth et al., 2024). For sample barcoding, cells were combined in pairs within each staining tube using CD45 labeled with 154Gd or 156Dy to distinguish the uninfected, *S.* Typhi-infected targets, media (PBMC cell alone) and SEB-stimulated samples. Cells were subsequently stained with cisplatin (194/195Pt) for viability assessment. After incubation with human Fc receptor-blocking IgG for 30 minutes, cells were stained for surface markers, fixed, permeabilized, and stained intracellularly using the 28-marker anti-human metal-labeled monoclonal antibody panel shown in **Supplementary Table 1**. Within 48 hours of labeling, cells were stained with an 191Ir/193Ir DNA intercalator, resuspended in EQ4 normalization beads (Standard BioTools), and acquired on a Helios mass cytometer (Standard BioTools). All mass cytometry experiments were performed at the Flow Cytometry and Mass Cytometry Core Facility of the University of Maryland School of Medicine Center for Innovative Biomedical Resources (CIBR), Baltimore, Maryland.

### 2.5 Data analysis

#### 2.5.1 Unsupervised data analysis

All mass cytometry data analyses were performed with FlowJo (v 10.8.1) and its plug-ins such as PeacoQC (v 1.5), Euclid (v 0.8), UMAP (v 3.1) and Phenograph (v 2.5). To ensure maximal data quality, the concatenated data was gated to exclude doublets, debris and calibration beads. Briefly, the onboard CyTOF software was used to normalize signals and convert data into the Flow cytometry standard (FCS) 3.0 format. FCS files were debarcoded and imported into FlowJo and transformed to arcsinh. Peak Extraction and Cleaning Oriented Quality Control (PeacoQC) plugin (FlowJo) was used to perform quality control on the data to evaluate the sample signal for regions of irregularity. PeacoQC Good Events were used for further analysis as described previously (Booth et al., 2024). Gates were generated to detect live CD3^+^ CD4^-^ CD8^+^ T cells single events which were down sampled according to standard guidelines for the assembly of datasets for multidimensional reduction analyses (Becht et al., 2018; Kobak and Berens, 2019) to 5000 events for each volunteers/time point and culture condition to ensure equal contribution of each sample, followed by file concatenation using FlowJo. Then, UMAP (Uniform Manifold Approximation and Projection; version 3.1) was used to perform dimensionality reduction as previously described (Becht et al., 2018) according to standard guidelines for dimensional reduction analysis (Becht et al., 2018; Ujas et al., 2023). UMAPs were created in FlowJo using the plugin UMAP v3.1 (Nearest Neighbors (NN) = 45; Minimal distance = 0.1). Unsupervised clustering was performed using PhenoGraph v2.5 according to standard guidelines to determine the optimal K value (K = 120) for clustering analysis of high-dimensional data (DiGiuseppe et al., 2018). Euclid was used for quality control of clustering results to identify possible over- or under-clustering by assessing cluster separation and compactness with determination of the Taylor Index (Total index=150). PhenoGraph clusters were then embedded and visualized using sUMAP (Weight = 0.1) to create a reference map of all automatically detected CD8^+^ T clusters. The UMAP was used to illustrate the distribution of the clusters within the CD8^+^ T cells and its memory subsets (T_EM_, T_CM_ and T_EMRA_) and to visualize the surface expression of markers in each PhenoGraph cluster as described previously (Booth et al., 2024). ClusterExplorer (v 3.0) and ViolinBox (v 5.1.11) were used to perform hierarchical clustering for each cluster at each time point in TD and NoTD participants and to generate bubble plots showing expressing of the markers respectively. For each specific cluster response, we calculated *S.* Typhi-specific cluster responses as net percentage of events (background after culture with uninfected cells were subtracted from the values obtained following culture with *S.* Typhi-infected targets). Marker Enrichment Modeling (MEM), an unbiased phenotyping method (Diggins et al., 2015; Diggins et al., 2017; Diggins et al., 2018), was used to determine the phenotype of each cluster of interest in our analysis.

#### 2.5.2 T-REX: Tracking responder expanding analyses

To identify regions of significant differences between TD and NoTD across study phases (baseline, post-Ty21a vac, Challenge, post -challenge), Tracking Responders Expanding (T-REX) analysis (Barone et al., 2021) was performed on the relevant subsets (CD8^+^ T_EM_, T_CM_ and T_EMRA_) and time-point comparisons. Regions exceeding the threshold (90%) for differential abundance between outcome groups were extracted and the expression level of measured markers shown by bubble plot followed by phenotypic characterization using MEM.

#### 2.5.3 Feature-ranking and classification analysis

To identify variables associated with classification of typhoid diagnosis status, feature-ranking analyses were performed using cluster frequencies and marker expression values to distinguish volunteers who developed typhoid disease (TD) from those who did not (NoTD) using the method described by Yount et al., with modification (Yount et al., 2025).

##### 2.5.3.1 Definition of cell subpopulations

Cell subpopulations were defined using two complementary approaches: manual gating and unsupervised clustering. Manual gating identified CD8^+^ T_CM_, T_EM_, and T_EMRA_ subsets. In parallel, unsupervised clustering was performed using PhenoGraph, which generated clusters designated C01-C17 (**Supplementary Figure 2**).

##### 2.5.3.2 Construction of the feature matrix

Feature-ranking analysis was performed to identify cluster frequency and marker expression-based variables associated with typhoid diagnosis status, comparing volunteers who developed typhoid disease (TD) with those who did not (NoTD). For each clustering strategy and each marker, the upper-quartile expression value (75th percentile) was calculated within each cluster or manually gated subpopulation. In addition, cluster frequency was calculated for each sample as the proportion of cells assigned to a given cluster. These measurements were used as candidate features for downstream classification analyses. The feature matrix used for random forest modeling consisted of upper-quartile expression values for each marker-cluster combination together with cluster frequency features.

##### 2.5.3.3 Feature filtering, transformation, and imputation

To reduce the influence of rare clusters, clusters with a mean frequency <0.1% across all samples were excluded from the analysis. For the retained clusters, expression features were set to missing in samples in which the corresponding cluster frequency was <0.1%. Missing values were then imputed using the mean value of the corresponding feature across all samples. Expression features with upper-quartile values <2 across samples were excluded as low-signal features. The remaining expression features were transformed using the arcsinh function with a cofactor of 1.0. CD45RA and CD62L, which were used to define the manually gated CD8^+^ T memory subsets, were excluded from the random forest analyses.

##### 2.5.3.4 Random forest classification and feature importance analysis

Analyses were performed separately for the background, immunization, and challenge phases. The background phase included samples collected on Day -28 under ST stimulation conditions. The immunization phase included ST-stimulated samples collected on Days -14 and 0. The challenge phase included ST-stimulated samples collected on Days 7, 14, and 28 (**Supplementary Figures 1**, **2**). For each phase, classification was performed independently using the Random Forest Classifier algorithm implemented in scikit-learn with default parameters. The input variables consisted of the processed expression and frequency features described above, and the outcome variable was TD versus noTD status. Feature importance was quantified using raw Gini importance (mean decrease in impurity) derived from the trained random forest models. To evaluate the contribution of individual clusters, raw Gini importance values were also summarized at the cluster level by calculating the median importance across all expression features associated with a given cluster, together with the corresponding cluster frequency feature. All analyses were performed independently for each study phase.

#### 2.5.4 Supervised analysis

Data was analyzed using FlowJo version 10.8.1 after exclusion of doublets, debris and calibration beads and QC as described above. CD8^+^ T-cell subsets were defined using an established CD45RA/CD62L gating strategy: naïve cells, CD45RA^+^CD62L^+^; T_CM_, CD45RA^-^CD62L^+^; T_EM_, CD45RA^-^CD62L^-^; and T_EMRA_, CD45RA^+^CD62L^-^. This classification has been used in previous human T-cell immunophenotyping studies, including our previous publications in this field, and was maintained here to permit direct comparison with our earlier studies of *S.* Typhi-specific systemic and intestinal immunity (Fresnay et al., 2016; Booth et al., 2017; Fresnay et al., 2017; Booth et al., 2019b; Booth et al., 2019c; Booth et al., 2024). CCR7 was included in the CyTOF panel and evaluated within each CD45RA/CD62L-defined subset as a complementary marker of memory differentiation and lymph node-homing potential. Singlet CD3^+^ CD4^-^ CD8^+^ T_EM_ cells were gated conventionally and expression of CXCR3, PD-1, IL-17A, CCR6, CD27, integrin α4β7 and co-expression of CD27, GzB and IL-17A or co-expression of CD27, CCR6 and integrin α4β7 were evaluated from NoTD and TD participants at all time points (D-28 to D28) following stimulation with uninfected and *S*. Typhi B-LCL targets. Similar supervised analyses were performed for CD8^+^ T_CM_ and T_EMRA_. *S.* Typhi-specific responses were calculated for each of the markers and their co-expression and expressed as net percentage of positive cells (background after culture with uninfected cells were subtracted from the values obtained following culture with *S.* Typhi-infected targets).

### 2.6 Statistical analysis

Data were analyzed using the statistical software GraphPad Prism™ version 7.0 (Graphpad, San Diego, CA, USA) package. Statistical differences in median values between two groups (e.g., TD vs NoTD, D0 vs D7, cluster 1 TD vs NoTD) were determined using two-sided Wilcoxon rank-sum tests (Mann-Whitney U tests). *P* values < 0.05 were considered significant. Consistent with recent recommendations by the American Statistical Association (ASA) (Wasserstein and Lazar, 2016; Yaddanapudi, 2016; Booth et al., 2019a), particularly when analyzing data sets with relatively low numbers of volunteers, we also indicate trends in the expression of markers or cytokine responses where appropriate when the statistical analyses yielded values within a 0.05 < p < 0.1 range. Receiver operating characteristic (ROC) analysis was used to assess discriminatory performance of selected clusters or marker combinations.

## 3 Results

Human immunity to *S.* Typhi involves complex coordination between humoral and cellular responses. CD8^+^ T cells have become increasingly implicated in protection from typhoid disease both at the intestinal and systemic sites following infection or Ty21a vaccination (Booth et al., 2017; Booth et al., 2019b; Booth et al., 2019c; Booth et al., 2019a; Booth et al., 2020; Booth et al., 2021). Despite these findings, immune programming and correlates of protection of CD8^+^ T cells in typhoid disease remain poorly defined. To address this gap, we performed high-dimensional CyTOF profiling of *S.* Typhi-responsive CD8^+^ T cells across the CHIM timeline, spanning baseline, post-Ty21a immunization, challenge, and post-challenge time points, and stratified participants by clinical outcome (NoTD vs TD).

### 3.1 Unsupervised analysis identifies phenotype structure across CD8^+^ T_M_

Using concatenated files from TD and NoTD participants at all time points (D-28 to D28) and following stimulation with either *S*. Typhi-infected (ST) or uninfected (NI) B-LCL (targets), we performed a detailed analysis of subpopulations gated on memory CD8^+^ T (T_M_) cells. Dimensionality reduction by UMAP and unsupervised clustering with PhenoGraph were used to identify 17 CD8^+^ T-cell clusters in the dataset (**Figure 1**). We then used this UMAP reference map to visualize and assess the distribution of activation, functional (e.g., cytokines), and homing markers (**Figures 1A–C**). For example, we observed that activation markers (CD69, CD27, PD-1 ICOS, CD154) were distributed in unique patterns across the 17 CD8^+^ T clusters on the UMAP (**Figure 1A**). Similar observations were made for cytokines/chemokines (**Figure 1B**) and for homing markers (**Figure 1C**). These data suggested that CD8^+^ T cell clusters have unique combinations of markers that could be important in either Ty21a vaccination or protection from wt *S*. Typhi challenge. As previously reported, CD8^+^ T cells contained naïve T cells (T_Naïve_) and three T_M_ subsets based on the expression of CD62L and/or CD45RA, namely T effector memory (T_EM_), T_EM_ expressing CD45RA (T_EMRA_), and T central memory (T_CM_). We applied this hierarchical analysis to the clustering analysis and determined that each cluster is present at defined frequencies among CD8^+^ T subsets (**Figure 1D**). For example, clusters 5, 8,15,17 were less frequent in CD8^+^ T_EM_ than in CD8^+^ T_EM_ and T_EMRA_ while clusters 2, 3 were less frequent in CD8^+^ T_CM_ (**Figure 1D**). Interestingly, cluster 11 was not present in CD8^+^ T_EMRA_ as compared to CD8^+^ T_EM_ and T_EMRA_ (**Figure 1D**). Although all three memory compartments contained heterogeneous states, the composition and phenotypic representation of the clusters differed among T_EM_, T_CM_, and T_EMRA_, supporting the need for subset-specific analysis.

**Figure 1:**
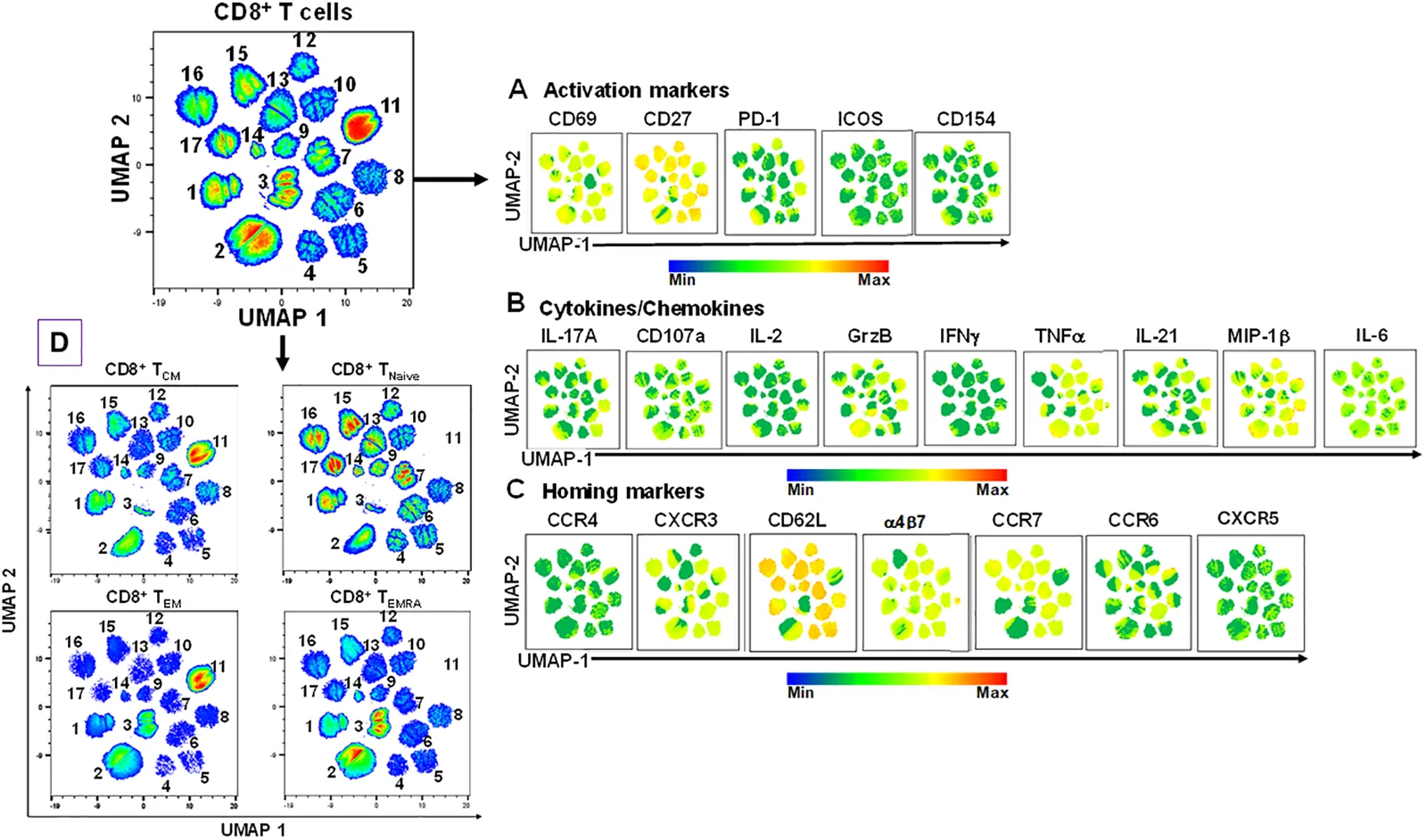
Unsupervised clustering and phenotype of TD and NoTD memory CD8^+^ T cells. Equal number of events from all participants (TD and NoTD) across all study timepoints (D-28 to D28) following stimulation with *S*. Typhi infected and uninfected targets were concatenated and dimensionality reduction analysis using Uniform Manifold Approximation and Projection (UMAP), and unsupervised clustering using Phenograph were performed to generate a reference map of all automatically detected CD8^+^ T cell clusters. The analysis identified 17 CD8^+^ T cell clusters spanning a range of frequencies for cluster abundance as shown by density plot. Using this reference UMAP, expression of **(A)** Activation markers (CD69, CD27, PD-1, ICOS, CD154), **(B)** Cytokines and chemokines (IL-17A, CD107a, IL-2, GranzymeB (GzB), IFNγ, TNFα, IL-21, MIP1β and IL-6), and **(C)** homing markers (CCR4, CXCR3, CD62L, integrin α4β7, CCR7, CCR6 and CXCR5) were evaluated. Scale bars indicate relative marker intensity. **(D)** Using classical markers of T memory (T_M_) subsets (i.e., CD62L and CD45RA), memory CD8^+^ T cells were identified and the distribution of the 17 clusters within each T_M_ subset calculated and shown for T central memory -T_CM_-; T effector memory-T_EM_-; T terminal effector memory RA^+^ -T_EMRA_- and T naïve CD8^+^ T cells.

### 3.2 TD and NoTD participants displayed distinct net *S*. *Typhi*-responsive CD8+ T_EM_ clusters

It is well established that *S.* Typhi-reactive CD8^+^ T_EM_ are elicited following Ty21a vaccination and after wt *S.* Typhi challenge in CHIM studies. However, the immune programming of CD8^+^T_EM_ in TD and NoTD participants remain incompletely understood. Thus, we measured whether CD8^+^T_EM_ clusters frequencies differed between TD and NoTD participants following stimulation with *S*. Typhi- infected and uninfected targets. Using ClusterExplorer, the frequencies of CD8^+^T_EM_ cluster 1-17 were assessed in TD and NoTD participants. We observed that various clusters were present at higher frequencies in TD than in NoTD participants as shown by the yellow-black color intensity scheme (**Supplementary Figure S3A**). For example, clusters 6, 7, 15 and 17 were more frequent in TD than in NoTD participants (**Supplementary Figure 3A**). These comparisons included all time points and stimulation conditions. Similarly, when CD8^+^ T_EM_ cluster frequencies were compared across stimulation conditions, some clusters (e.g., clusters 4, 6, 15-17) were present at higher frequencies following stimulation with *S.* Typhi-infected targets (ST) than with uninfected targets (NI) (**Supplementary Figure 3B**). These comparisons included all time points and participants.

To determine how S. Typhi-specific CD8^+^ T_EM_ clusters vary between TD and NoTD over time, we calculated net *S*. Typhi-specific clusters frequencies by subtracting background frequencies obtained with NI from those obtained with ST. The data were deconvoluted according to the time points (D-28 to D28) to evaluate the effects of Ty21a vaccination and wt *S*. Typhi challenge (**Figure 2**; **Supplementary Figure 3**). We then compared the frequencies of each *S*. Typhi-specific cluster (Levine et al., 1987; Simanjuntak et al., 1991; Levine et al., 1999; Salerno-Goncalves et al., 2002; Salerno-Goncalves et al., 2004; Salerno-Goncalves et al., 2005; Salerno-Goncalves et al., 2010; Waddington et al., 2014; Klemm et al., 2018; Shakya et al., 2019; Jamilah et al., 2020; Patel et al., 2021; Qadri et al., 2021; Shakya et al., 2021; Sztein and Booth, 2022; Batool et al., 2024; Neuzil et al., 2024) gated on either TD or NoTD at each time point (**Figure 2**; **Supplementary Figure S4**). These values were expressed as net percentages of events. Net frequencies of *S.* Typhi-responsive CD8+ T_EM_ clusters 1-17 were then assessed longitudinally in individual TD (blue line) and NoTD (red line) participants, with median responses shown (**Figure 2**; **Supplementary Figure 4**). Furthermore, Receiver operating characteristic (ROC) curves were used to discriminate statistically (area under the curve [AUC] and p value) between NoTD and TD participants. In addition, we also determined the phenotype of each cluster using the marker enrichment modeling (MEM) which assigns a score of -10 to 10 for each marker to quantify the extent to which a marker is enriched (positive score) and under-expressed (negative score) in a given cluster relative to a reference cell population.

**Figure 2:**
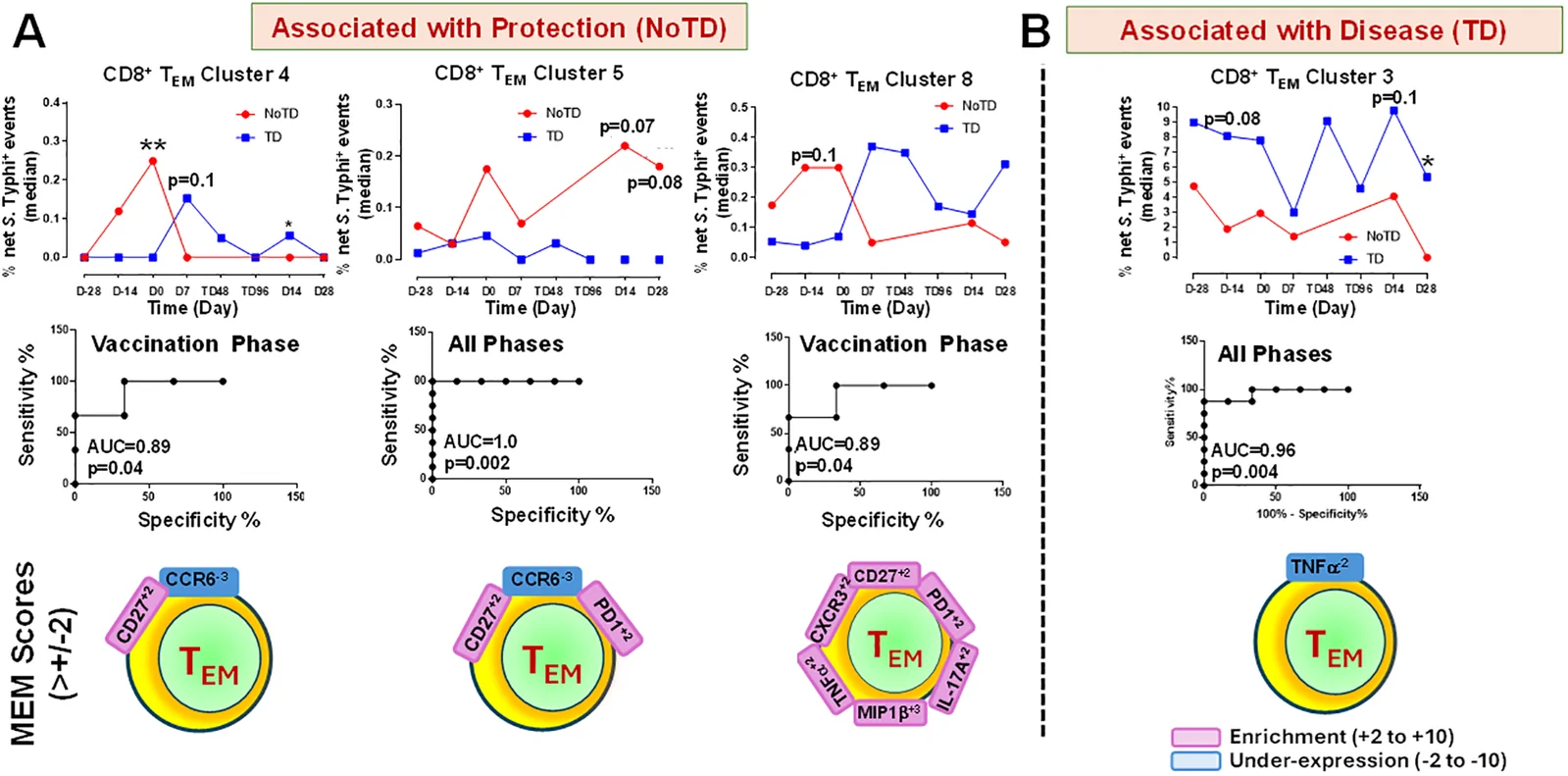
Distinct *S*. Typhi-specific CD8^+^ T_EM_ clusters discriminate protected (NoTD) from typhoid disease (TD) outcomes. The net *S*. Typhi responses of CD8^+^ T_EM_ clusters were calculated as described in methods in PBMC samples from participants in both TD and NoTD groups at all time points (D-28 to D28). *S*. Typhi-specific CD8^+^ T_EM_ associated with **(A)** Protection and **(B)** Susceptibility (Disease) were identified (NoTD (Red); TD (Blue)). Receiver operating characteristic (ROC) curves summarize discriminatory performance (area under the curve [AUC] and p-value) between NoTD and TD groups during the phase as indicated. Marker Enrichment Modeling (MEM) signatures for each cluster are shown highlighting enriched (MEM + 2 to +10; pink) and under-expressed (MEM −2 to −10; blue) features. Significant differences between TD and NoTD volunteers at each time points are represented by ** p<0.005. P-values are indicated for trends. Values are shown as median % of events.

Interestingly, out of the 17 clusters (**Figure 2**; **Supplementary Figure 4**), three *S.* Typhi-specific CD8^+^ T_EM_ clusters (cluster 4, 5 and 8) were associated with protection (**Figure 2A**). Net *S*. Typhi-specific Cluster 4 in NoTD group showed increasing frequencies following vaccination with statistically significant differences (p<0.05) at D0 as compared to TD. This response declines following wt *S*. Typhi challenge (**Figure 2A**). ROC analysis identified a significant difference (p=0.04) between NoTD and TD participants during the vaccination phase for cluster 4 (**Figure 2A**). MEM analysis showed that CD8^+^ T_EM_ cluster 4 was enriched for CD27 but under-expressed CCR6 (**Figure 2A**). Similarly, the frequencies of net *S*. Typhi-specific CD8^+^ T_EM_ cluster 5 were higher in NoTD than TD at most time points with significant differences observed between the two groups (p=0.002) using ROC (**Figure 2A**). In addition, the phenotype of cluster 5 was similar to cluster 4 with the addition of enrichment in PD-1 (**Figure 2A**). Finally, net *S*. Typhi-specific CD8^+^ T_EM_ Cluster 8 frequencies were higher in NoTD than TD at baseline and following Ty21a vaccination (**Figure 2A**). ROC analysis displayed significant differences (p=0.04) in the vaccination phase between NoTD and TD group (**Figure 2A**). Notably, the CD8^+^ T_EM_ cluster 8 displayed strong enrichment for effector molecules, including IL-17A, MIP-1β, TNFα, and CXCR3 (**Figure 2A**).

In contrast, out of the 17 clusters, we observed one CD8^+^ T_EM_ cluster that was associated with the development of typhoid disease. Net *S*. Typhi-specific CD8^+^ T_EM_ cluster 3 frequencies were higher in TD than NoTD across all time points and significantly discriminated the two groups across phases by ROC analysis (p = 0.004) (**Figure 2B**). The MEM phenotype of CD8^+^ T_EM_ cluster 3 showed an under-expression of TNFα (**Figure 2B**). Collectively, these findings identify *S.* Typhi-specific CD8^+^ T_EM_ clusters which are either associated with protection or susceptibility to typhoid disease. These clusters displayed unique phenotypic signatures and differential longitudinal kinetics between NoTD and TD individuals, highlighting their potential as immunological correlates of protection in the context of oral vaccination and wt *S*. Typhi infection. These data support a model in which protection is not explained by a single marker, but rather by coordinated effector and trafficking markers within T_EM_ clusters.

### 3.3 Phase-specific divergence of TD and NoTD CD8^+^ T_EM_ memory programs.

We next used Tracking Responders Expanding (T-REX) FlowJo plugin which identifies local cellular neighborhoods within a UMAP embedding of concatenated events that can pinpoint significant differences in responding immune cells subpopulation (e.g., CD8^+^ T_EM_) between two conditions (e.g., TD vs NoTD), thereby revealing cell populations that differ in abundance between the groups.

Regions of significant differences are then analyzed to identify cell types, marker expression by bubble plots and key features using MEM. Thus, concatenated files using the same number of events from all the TD and NoTD participants at all time points (D-28 (8TD and 8NoTD), D0 (7TD and 6NoTD), D7 (5TD and 6NoTD) and D28 (6TD and 7NoTD) stimulated with *S*. Typhi-infected (ST) B-LCL were used to detect significant differences in responding CD8^+^ T_EM_ between TD and NoTD participants at baseline (D-28), following Ty21a vaccination (D0), D7 and D28 after *S*. Typhi challenge. Interestingly, at baseline (D-28), T-REX revealed two regions of significant change (≥90%) containing clusters (50 events or more) for NoTD (red) and TD (blue) (**Figure 3A**). These NoTD and TD CD8^+^ T_EM_ clusters displayed distinct and contrasting levels of expression of activation, functional and homing markers as shown in the bubble plots (**Figure 3A**). For example, CXCR3 and IL-17A are expressed higher in CD8^+^ T_EM_ NoTD than TD participants at baseline while CCR6 was increased in CD8^+^ T_EM_ TD compared to NoTD participants (**Figure 3A**). We next assessed MEM scores and observed contrasting phenotypes in CD8^+^ T_EM_ between NoTD and TD groups. The phenotype of CD8^+^ T_EM_ in the NoTD group is enriched with multiple effector markers (e.g., IL-17A, MIP1β, TNFα, IL-21, PD-1, CXCR5, CD154) while in the TD group, the phenotype showed under-expression of effector responses (e.g., IL-21, IL-17A, CXCR3) but enriched for homing (integrin α4β7, CCR6) and activation (CD69) markers (**Figure 3A**). Following Ty21a vaccination, T-REX analysis similarly showed various regions of significant change (≥90%) containing clusters (50 events or more) for NoTD (red) and TD (blue) (**Figure 3B**). These NoTD and TD CD8^+^ T_EM_ clusters displayed distinct and contrasting levels of expression of activation, functional and homing markers as shown in bubble plot (**Figure 3B**). For example, CXCR3, CD107a, PD-1, MIP1β, IL-21 and GzB were expressed at higher levels in CD8^+^ T_EM_ NoTD than in TD participants following Ty21a vaccination while CCR6 and integrin α4β7 were increased in CD8^+^ T_EM_ TD compared to NoTD participants (**Figure 3B**). The phenotype as determined by MEM score showed that CD8^+^ T_EM_ NoTD group is enriched with multiple effector markers (e.g., MIP1β, TNFα, IL-21, PD-1, GzB) while TD group phenotype under-expressed effector responses (e.g., IL-21, IL-17A, CXCR3, GzB) but is enriched for homing (integrin α4β7 and CCR6) markers following Ty21a vaccination (**Figure 3B**).

**Figure 3:**
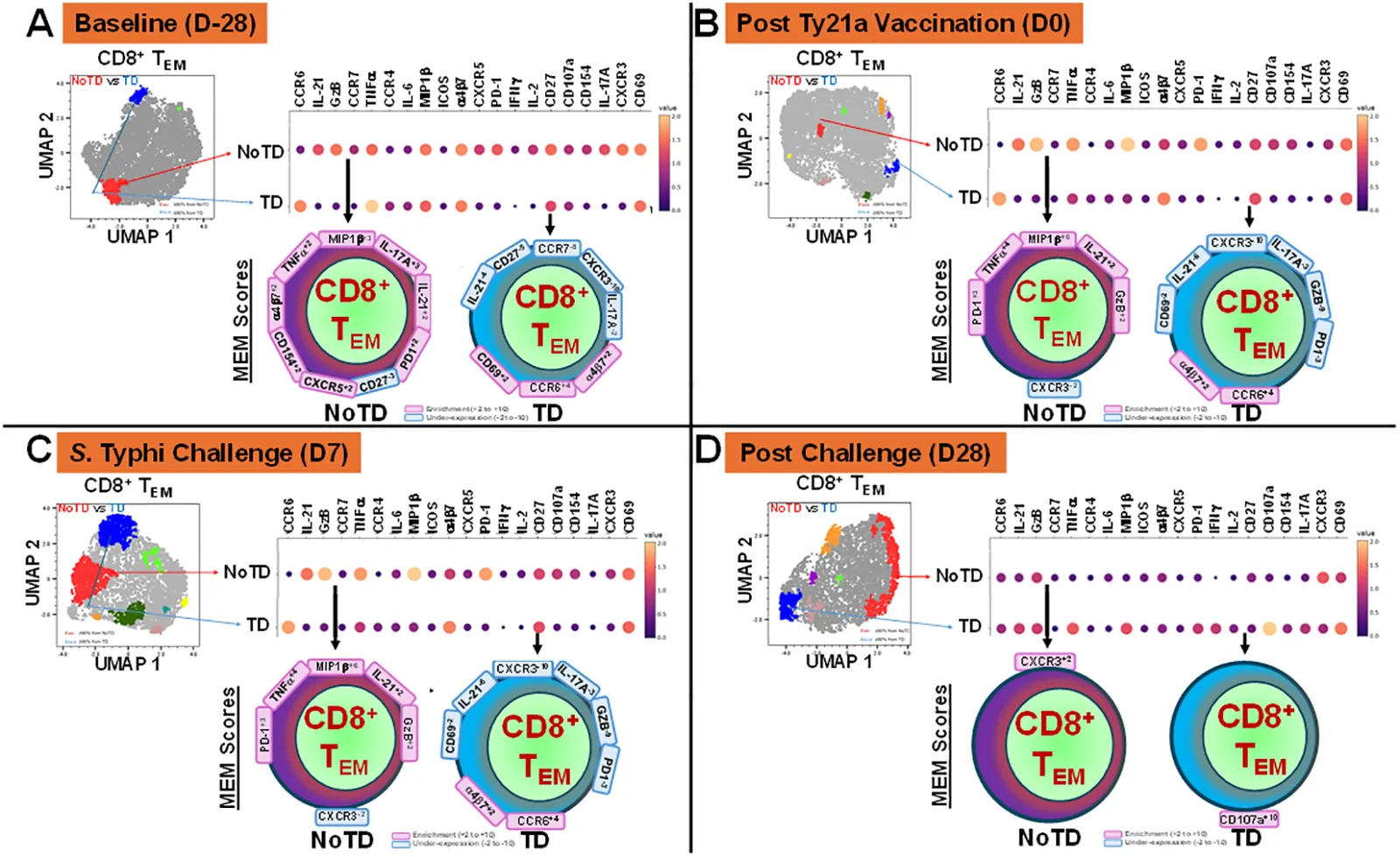
Phase-specific T-REX analysis distinguishes TD vs NoTD CD8^+^ T_EM_ immunophenotypes. Tracking Responders Expanding (T-REX) analysis was performed at each time point representing the different phases of the study to compare NoTD vs TD at **(A)** baseline (D-28), **(B)** post Ty21a vaccination (D0), **(C)** post *S*. Typhi challenge (D7), and **(D)** day 28 post-challenge (D28) in CD8^+^ T_EM_ stimulated with *S*. Typhi-infected targets. Regions/clusters showing >90% difference were identified as unique to TD (blue) and NoTD (red). Dot/bubble summaries depict relative expression of measured markers in NoTD vs TD for the highlighted regions (color intensity corresponds to scaled marker level). MEM score thresholds shown in legend.

Following the *S*. Typhi challenge at D7, we observed various regions of significant change (≥90%) containing clusters (50 events or more) for NoTD (red) and TD (blue) (**Figure 3C**). The expression of markers again was dissimilar, with NoTD and TD CD8^+^ T_EM_ clusters displaying distinct levels of activation, functional, and homing markers as shown in bubble plot (**Figure 3C**). For example, CD8^+^ T_EM_ from TD group showed a less activated phenotype with cytokines/chemokines such as MIP1β, TNFα, IL-21, GzB and CD107a being expressed at lower levels than in NoTD participants following wt *S*. Typhi challenge (**Figure 3C**). The phenotype as determined by MEM score showed that CD8^+^ T_EM_ NoTD group is enriched with multiple effector markers (e.g., MIP1β, TNFα, IL-21, PD-1, GzB) while the TD group under-expressed effector responses (e.g., IL-21, CXCR3, GzB, PD-1) but is enriched for homing (integrin α4β7, CCR6) markers following wt *S*. Typhi challenge (**Figure 3C**). Finally, at D28, we observed a few regions of >90% difference between TD and NoTD (**Figure 3D**). Interestingly, as shown in the bubble plots, the expression marker profile of TD participants is different than that of NoTD (**Figure 3D**). For example, TD CD8^+^ T_EM_ displayed higher levels of CD107a, IL-21, IFNγ, PD-1, MIP1β and TNFα than in NoTD participants post-challenge (D28) (**Figure 3D**).

MEM analysis showed that CD8^+^ T_EM_ in the NoTD group is downregulated as it is only enriched in expression of CXCR3 while the TD group displayed an enrichment in the cytotoxic marker CD107a (**Figure 3D**) following post-challenge at D28. Collectively, these phase-specific T-REX analyses delineate divergent immunophenotypic trajectories of CD8^+^ T_EM_ between TD and NoTD individuals, highlighting potential immune correlates of protection versus susceptibility in the context of oral vaccination and *S.* Typhi challenge.

### 3.4 Feature-ranking analysis converges on recurrent markers associated with clinical outcome

Building on the CHIM framework and immunomonitoring strategies previously used to investigate outcome-associated cellular immunity in typhoid challenge studies (McArthur et al., 2015; Fresnay et al., 2016; Fresnay et al., 2017), we applied an unbiased clustering approach to resolve discrete CD8^+^ T-cell states and to prioritize outcome-associated populations and markers through cluster-level enrichment and classifier-based feature ranking. To identify a concise set of features contributing to clinical outcome discrimination, we applied feature-ranking analysis using cluster frequencies and marker expression values across study phases. This approach highlighted a limited number of markers that repeatedly contributed to TD versus NoTD separation. We used Gini importance scores to quantify the predictive value of each immune feature. High Gini scores indicate strong discriminators between clinical outcomes, helping us prioritize markers linked to protection or susceptibility. Following feature ranking analysis, we identified four top clusters (Cluster 2, 5, 7 and 13) based on the median Gini importance of all features of each cluster (**Figure 4**; **Supplementary Figure 5**). For each cluster (cluster 2, 5, 7, 13), the Gini score for the features that show significantly differential expression between TD vs NoTD (Wilcoxon rank-sum tests p < 0.1) were assessed across each study phase: baseline, immunization, and challenge. The blue bars represent specific features associated with development of typhoid disease (TD) while the red bars represent features that are associated with protection (NoTD) (**Figure 4**). For example, CD27 is predictive of susceptibility as it is associated with TD in all clusters and across all phases (**Figure 4**). In contrast, IL-17A is predictive of protection as it is associated with NoTD particularly in the challenge phase within all the clusters (**Figure 4**). In addition, we noted that among the most recurrent NoTD-associated features were MIP1β and CD107a (Cluster 7 & 8), CXCR5 (cluster 7) and the frequency of cluster 5 (Freq) whereas markers including GrandzymeB (GzB) (cluster 5,7 & 13), and integrin α4β7 (all clusters) contributed strongly to TD-associated profiles (**Figure 4**). These analyses also provided a bridge between cluster-level discovery and biologically interpretable marker programs. Altogether these complementary analytic methods reveal divergent immune response signatures following Ty21a vaccination and wt *S*. Typhi challenge. Notably, NoTD signature immune programs include CXCR5, IL-17A, CXCR3, TNFα, PD-1 and IL-21.

**Figure 4:**
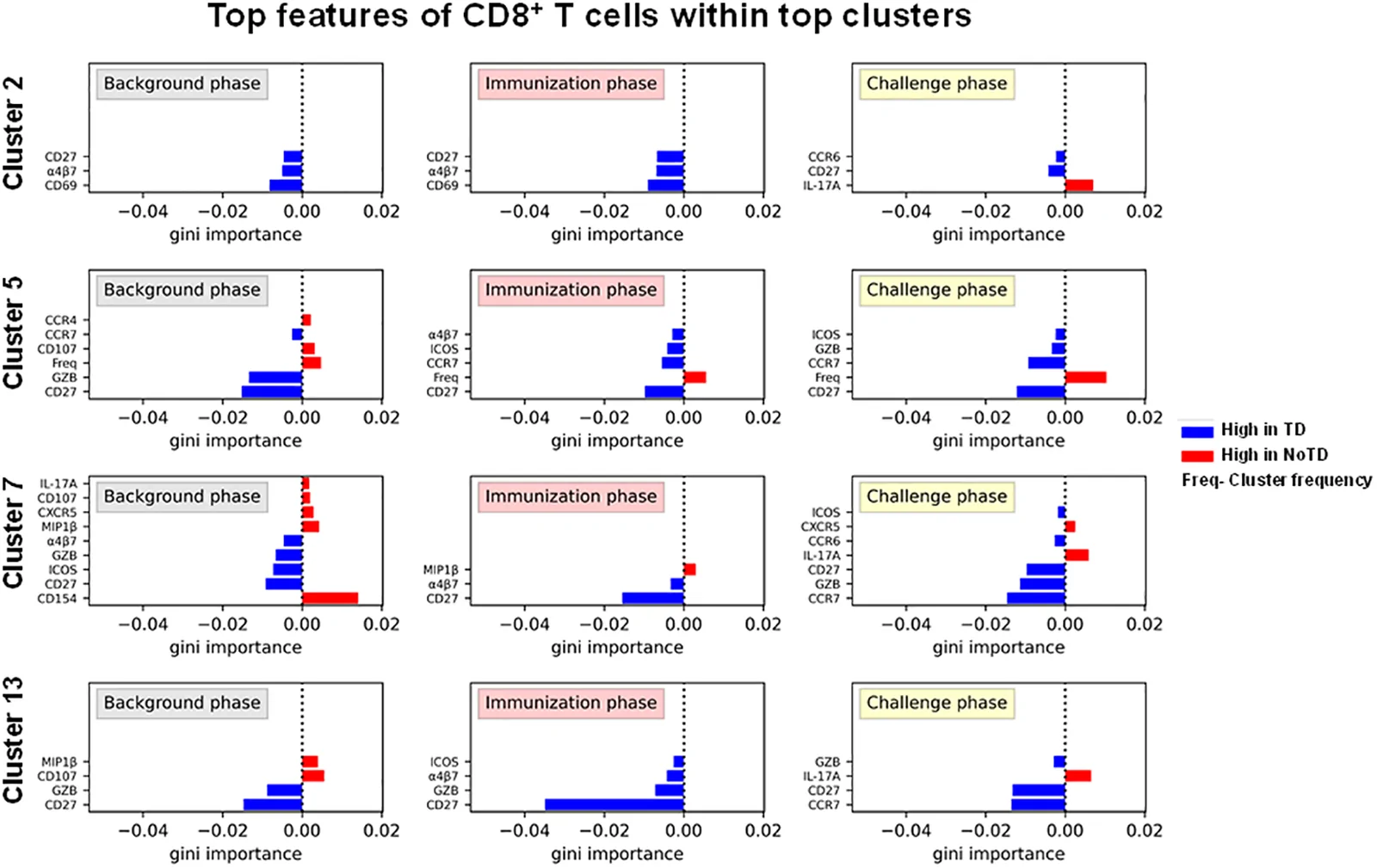
Machine-learning feature ranking identification of CD8^+^ T cells cluster- and phase-specific predictors of clinical outcome. Gini importance was used from trained models to quantify the predictive value of each immune feature. We evaluated the Gini importance score in the 4 top rank clusters (cluster 2, 5, 7, 13) across each study phase: baseline, immunization, and challenge. The blue bars represent specific features associated with development of typhoid disease (TD) while the red bars represent features that are associated with protection (NoTD). Freq represents the frequency of CD8^+^T cells from the cluster.

### 3.5 Validation of signature markers associated with clinical outcome for CD8^+^ T_EM_

Next, we validated our unsupervised findings using traditional supervised gating approaches on ex-vivo CD8^+^ T_EM_ PBMC obtained from NoTD and TD participants across all study time points. Remarkably, the signature markers of CD8^+^ T_EM_ associated with protection such as CXCR3 (baseline and Ty21a vaccination), PD-1 and IL-17A (D7 after S. Typhi challenge) are significantly higher in NoTD than in TD participants (**Figures 5A–C**). CD8^+^ T_EM_ cells that express CD27^-^GzB^-^IL-17A^+^ are significantly higher in NoTD than TD participants at D7 and D14 following *S*. Typhi challenge (**Figure 5D**). In contrast, signature markers associated with susceptibility were found to be significantly higher in TD than in NoTD participants (**Figures 5E–H**).

**Figure 5:**
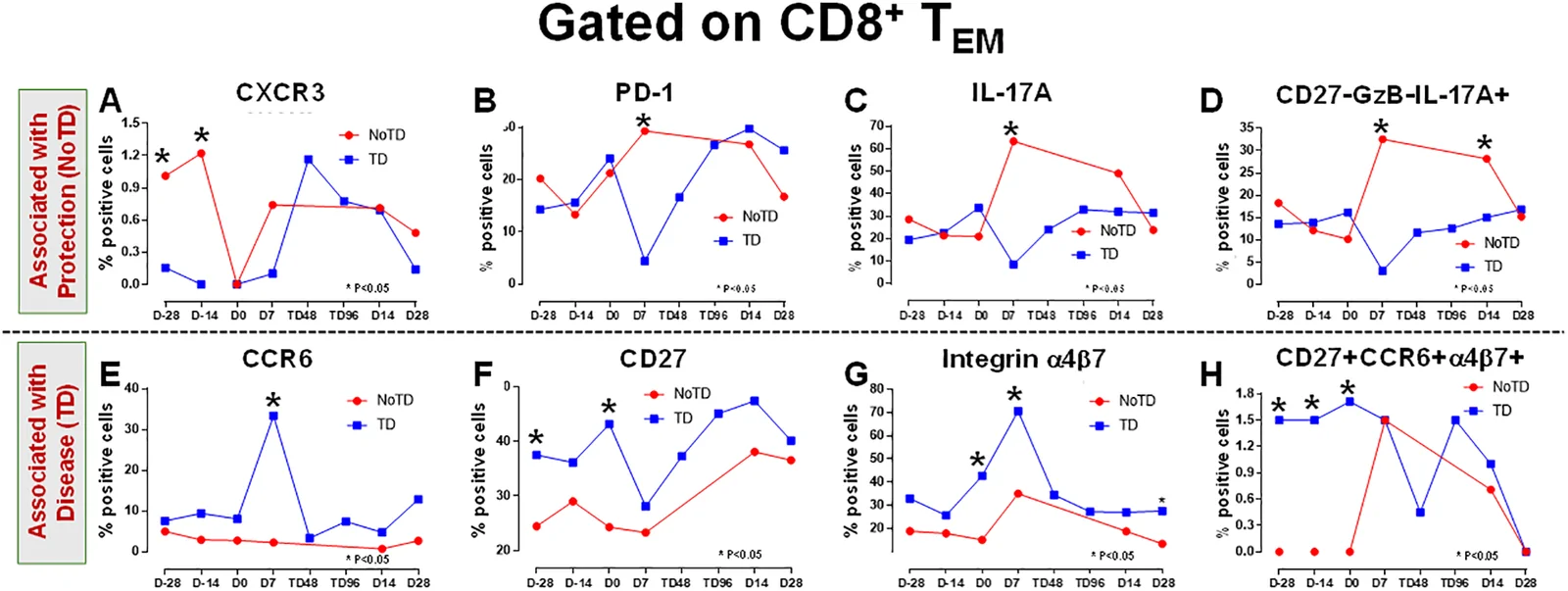
Validation of clinical outcome-associated CD8^+^ T_EM_ signature markers. Using CD8^+^ T_EM_ cell subsets obtained ex-vivo PBMC from participants in both TD and NoTD groups at all time points (D-28 to D28), conventional supervised analysis was performed to determine signature markers associated with TD and NoTD. CD8^+^ T_EM_ markers associated with protection (NoTD), **(A)** CXCR3, **(B)** PD-1, **(C)** IL-17A and **(D)** CD27^-^GzB^-^IL-17A^+^ and typhoid disease, **(E)** CCR6, **(F)** CD27, **(G)** Integrin α4β7 and **(H)** CD27^+^ CCR6^+^ α4β7^+^ were determined and compared between TD and NoTD across all time points in the study. Significant differences between TD and NoTD volunteers at each time points are represented by *p<0.05.

Markers such as CCR6 (D7 after challenge), CD27 (Baseline and after Ty21a vaccination) and integrin α4β7 (After Ty21a vaccination and D7 after *S*. Typhi challenge) are significantly higher in TD than NoTD participants (**Figures 5E–G**). CD8^+^ T_EM_ gated on CD27^+^CCR6^+^ α4β7^+^ are significantly higher in TD than NoTD participants at baseline and following Ty21a vaccination (D-14 and D0) (**Figure 5H**). Thus, this cross-validation provides greater confidence in our discovery of immune signatures predictive of protection or susceptibility.

### 3.6 CD8^+^ T_CM_ unsupervised analysis and phenotype structure

We next used the UMAP reference map generated above that was gated on CD45RA^-^ CD62L^+^ CD8^+^ T cells, representing central memory T cells (CD8^+^ T_CM_) to visualize and assess the distribution of activation, functional (e.g., cytokines), and homing markers (**Figures 6A–C**). CD8^+^ T_CM_ clusters displayed partially overlapping but distinct marker combinations consistent with central memory biology (**Figures 6A–C**). For example, we observed that activation markers (CD69, CD27, PD-1, ICOS, CD154) were distributed in unique patterns across the 17 clusters on the UMAP (**Figure 6A**). Similar observations were made for cytokines/chemokines (**Figure 6B**) and for homing markers particularly CCR7 (**Figure 6C**). These data suggested that CD8^+^ T_CM_ clusters have unique combinations of markers that could be important in either Ty21a vaccination or protection from wt *S*. Typhi challenge.

**Figure 6:**
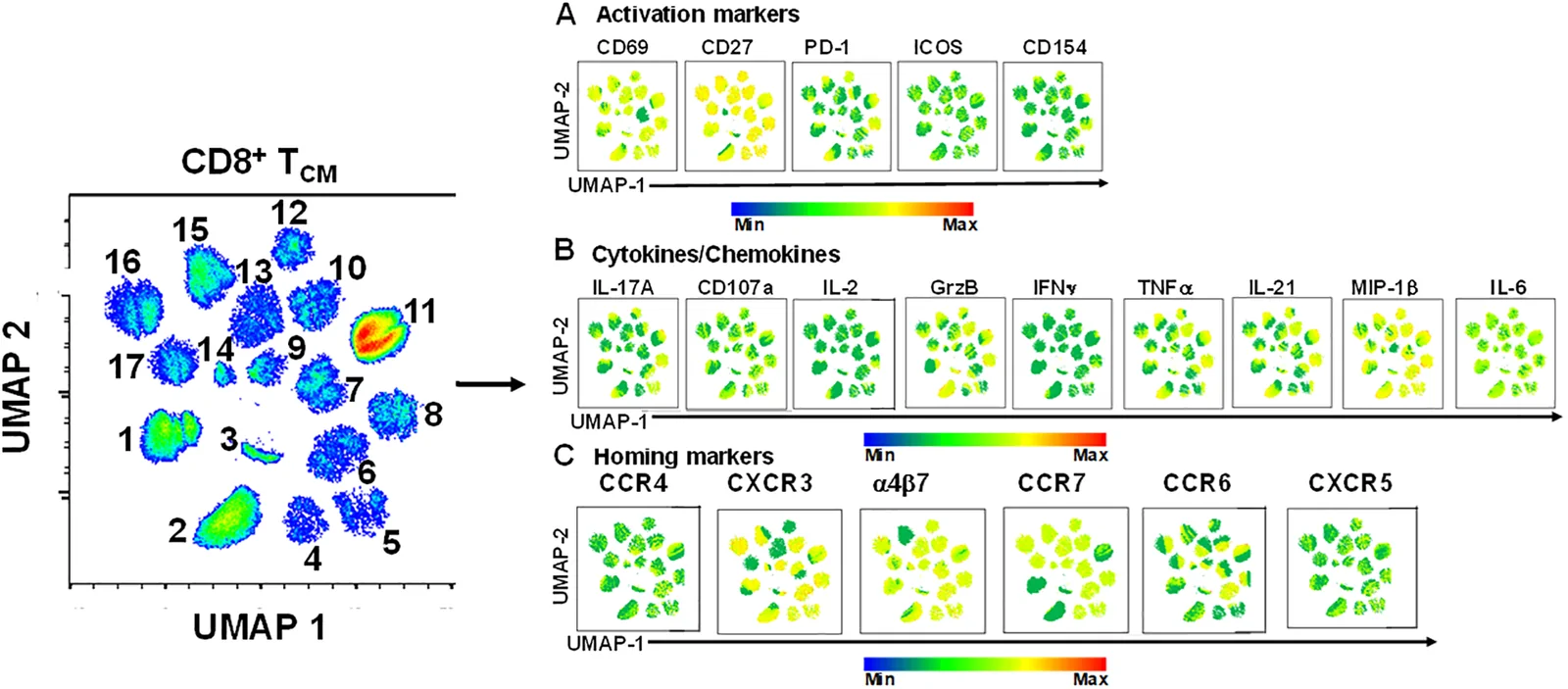
Unsupervised clustering and phenotype of TD and NoTD memory CD8^+^ T_CM_. The distribution of memory CD8^+^ T_CM_ subset resulted in 17 clusters. **(A)** Activation markers (CD69, CD27, PD-1, ICOS, CD154), **(B)** Cytokines and Chemokines (IL-17A, CD107a, IL-2, GranzymeB (GrzB), IFNγ, TNFα, IL-21, MIP1β and IL-6), and **(C)** homing markers (CCR4, CXCR3, CD62L, integrin α4β7, CCR7, CCR6 and CXCR5) were evaluated in the 17 CD8^+^ T_CM_ cell clusters. Scale bars indicate relative marker intensity.

### 3.7 *S*. Typhi-responsive CD8^+^T_CM_ identify outcome-linked clusters across study phases

Our group have previously shown direct evidence that CD8^+^ T_CM_ participate in the vaccine-induced anti-*S.* Typhi responses following oral Ty21a vaccination by inducing IFN-γ-secreting CD8^+^ T_CM_ with gut- and lymphoid tissue–homing potential (Wahid et al., 2015). However, the immune programming of CD8^+^ T_CM_ in TD and NoTD participants remains incompletely understood. Thus, we determined whether CD8^+^ T_CM_ cluster frequencies differed between TD and NoTD participants following stimulation with *S*. Typhi-infected and uninfected targets. Using ClusterExplorer, the frequencies of CD8^+^T_CM_ cluster 1-17 were assessed in TD and NoTD participants. We observed that there were various clusters present at higher frequencies in NoTD than in TD participants as shown by the yellow color intensity (**Supplementary Figure 6A**). For example, clusters 5, 8 and 12 were more frequent in NoTD than in TD participants (**Supplementary Figure 6A****).** However, these comparisons included all time points and stimulation conditions. Similarly, when CD8^+^ T_CM_ cluster frequencies were compared across stimulation conditions, some clusters (e.g., clusters 5, 8, 9 and 16) were present at higher frequencies following stimulation with *S.* Typhi-infected targets (ST) than with uninfected targets (NI) (**Supplementary Figure S6B**). However, these comparisons included all time points and participants. Thus, to interpret how *S*. Typhi-specific CD8^+^ T_CM_ clusters varies between TD and NoTD over time, we deconvoluted the data as shown above for CD8^+^ T_EM_ to evaluate the effects of Ty21a vaccination and wt *S*. Typhi challenge. We then compared the frequencies of each *S*. Typhi-specific CD8^+^ T_CM_ cluster (Levine et al., 1987; Simanjuntak et al., 1991; Levine et al., 1999; Salerno-Goncalves et al., 2002; Salerno-Goncalves et al., 2004; Salerno-Goncalves et al., 2005; Salerno-Goncalves et al., 2010; Waddington et al., 2014; Klemm et al., 2018; Shakya et al., 2019; Jamilah et al., 2020; Patel et al., 2021; Qadri et al., 2021; Shakya et al., 2021; Sztein and Booth, 2022; Batool et al., 2024; Neuzil et al., 2024) on either TD or NoTD participants at each time point (**Figure 7**; **Supplementary Figure 7**). Next, we asked whether net *S.* Typhi-responsive CD8^+^ T_CM_ clusters differed by clinical outcome across the study period. Interestingly, out of the 17 clusters, we observed that there were two *S.* Typhi-specific CD8^+^ T_CM_ clusters (clusters 5 and 12) that were associated with protection (**Figure 7A**). *S*. Typhi-specific Cluster 5 showed higher frequencies in NoTD (red line) than in TD (blue line) participants at all time points albeit with no statistical significance at any time point (**Figure 7A**).

**Figure 7:**
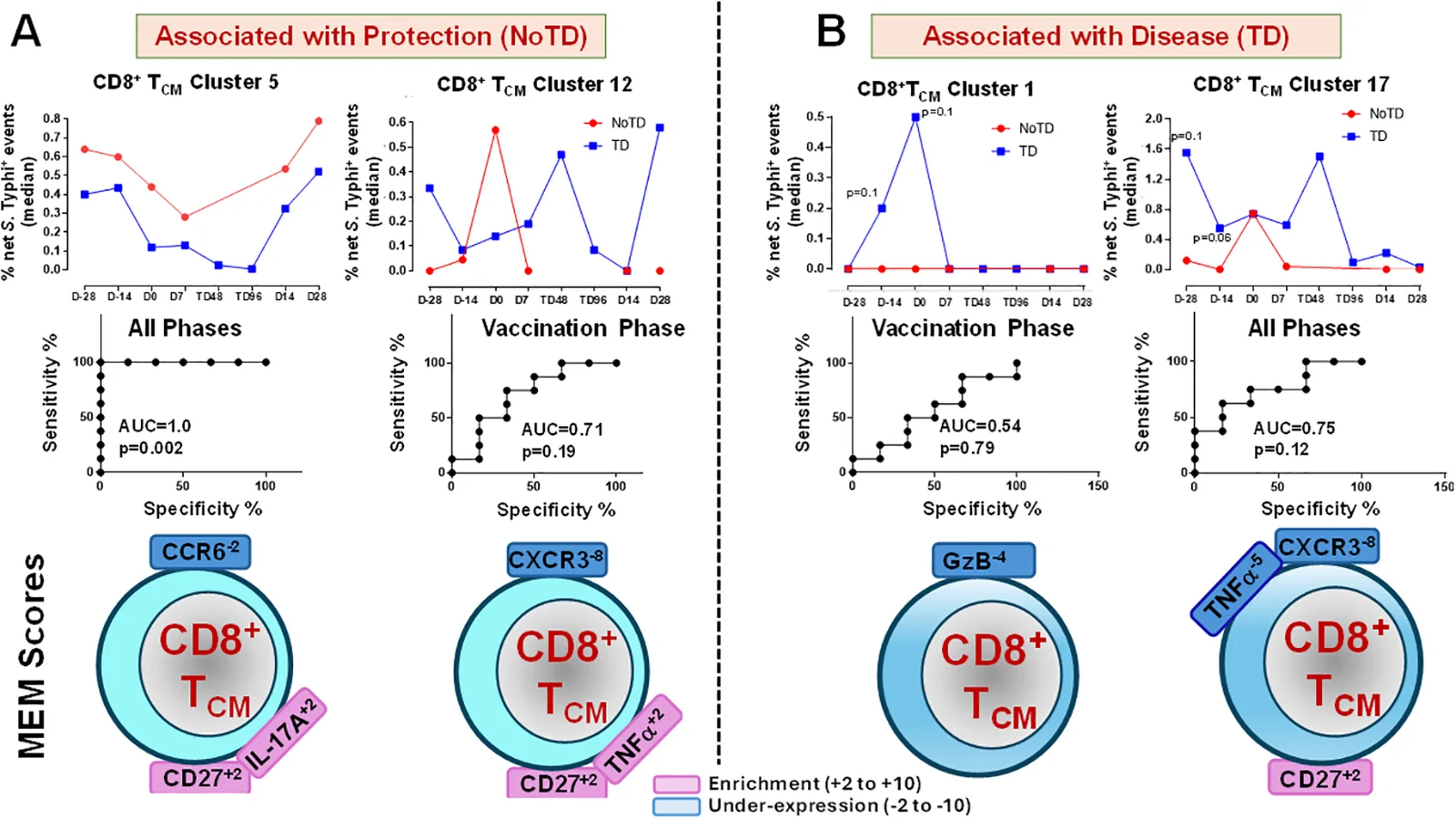
*S*. Typhi-specific CD8^+^ T_CM_ clusters distinguished protected (NoTD) from typhoid disease (TD) outcomes. Net *S*. Typhi responses of CD8^+^ T_CM_ clusters were determined in PBMC samples from participants in both TD and NoTD groups at all time points (D-28 to D28). *S*. Typhi-specific CD8^+^ T_CM_ associated with **(A)** protection and **(B)** susceptibility (Disease) were examined (NoTD (Red); TD (Blue)). Receiver operating characteristic (ROC) curves summarize discriminatory performance (area under the curve [AUC] and p-value) between NoTD and TD groups during the phase as indicated. Marker Enrichment Modeling (MEM) signatures for each cluster are shown highlighting enriched (MEM + 2 to +10; pink) and under-expressed (MEM −2 to −10; blue) features. Significant differences between TD and NoTD participants at each time points are represented by * p<0.05. P-values are indicated for trends Values are shown as median % of events.

Using ROC, we observed significant (p<0.05) statistical differences between NoTD and TD group across all phases of the study (**Figure 7A**). Next, cluster 5 was phenotype by MEM and we observed that it is enriched for CD27 and IL-17A but under-expressed CCR6 (**Figure 7A**). Similarly, the frequencies of *S*. Typhi-specific cluster 12 were observed to be higher in NoTD than TD participant following Ty21a vaccination (D0) with no significant differences observed between the two groups using ROC (**Figure 7A**). The phenotype of cluster 12 as determined by MEM displayed an enrichment in CD27 and TNFα but an under-expression of CXCR3 (**Figure 7A**). In contrast, out of the 17 clusters, we also observed CD8^+^ T_CM_ cluster 1 and 17 were associated with susceptibility to typhoid disease (**Figure 7B**).

Net *S*. Typhi-specific Cluster 1 exhibited increasing frequencies in TD but not NoTD participants following Ty21a vaccination, which showed no significance between the two groups as analyzed by ROC (**Figure 7B**). The phenotype of cluster 1 showed an under-expression of GzB in this CD8^+^ T_CM_ cluster (**Figure 7B**). Similar assessment of cluster 17 revealed that net *S.* Typhi-specific cluster was higher across all time points in TD than in NoTD participants (**Figure 7B**). However, ROC determination showed no significant differences between the two groups (**Figure 7B**). CD8^+^ T_CM_ Cluster 17 phenotype showed enrichment of CD27 but under-expression of TNFα and CXCR3 (**Figure 7B**). Collectively, these findings identify net *S. Typhi*-specific CD8^+^ T_CM_ clusters which are either associated with protection or susceptibility to typhoid disease. These clusters have unique phenotypic signatures and differential kinetics between NoTD and TD participants. These results suggest that *S.* Typhi-specific CD8^+^ T_CM_ programs may contribute or reflect immune readiness that shapes the downstream CD8^+^ responses to challenge.

### 3.8 T-REX analysis uncovers phase-specific divergence of TD and NoTD CD8^+^ T_CM_ immune programs

As described previously, we concatenated files using the same number of events from CD8^+^ T_CM_ obtained from all TD and NoTD participants at all time points following stimulation with *S*. Typhi-infected (ST) B-LCL to detect significant differences in responding CD8^+^ T_CM_ between TD and NoTD participants at D-28, D0, D7 and D28. Interestingly, at D-28, T-REX revealed two regions of significant change (≥90%) containing CD8^+^ T_CM_ clusters for NoTD (red) and TD (blue) among others (**Figure 8A**). These NoTD and TD CD8^+^ T_CM_ clusters displayed distinct and contrasting levels of expression of activation, functional and homing markers as shown in the bubble plots (**Figure 8A**).

**Figure 8:**
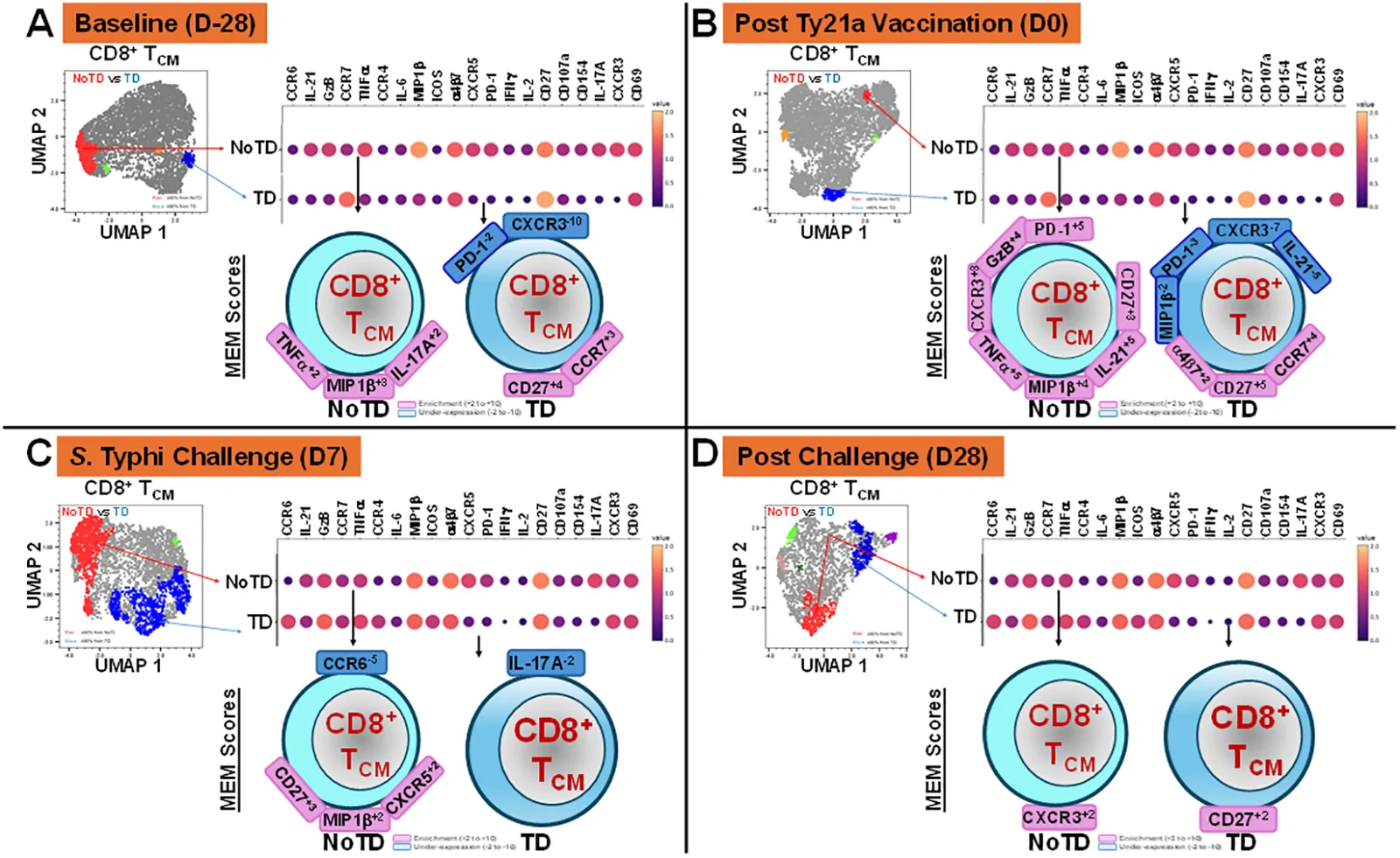
Distinct phase-specific TD vs NoTD CD8^+^ T_CM_ signature program using T-REX analysis. Tracking Responders Expanding (T-REX) analysis was performed at each time point representing the different phases of the study to compare NoTD vs TD at **(A)** baseline (D-28), **(B)** post Ty21a vaccination (D0), **(C)** post *S*. Typhi challenge (D7), and **(D)** day 28 post-challenge (D28) in CD8^+^ T_CM_ stimulated with *S*. Typhi-infected targets. Regions/clusters showing >90% difference were identified as unique to TD (blue) and NoTD (red). Dot/bubble summaries depict relative expression of measured markers in NoTD vs TD for the highlighted regions (color intensity corresponds to scaled marker level). MEM score thresholds shown in legend.

For example, CXCR3, CXCR5, and IL-17A in CD8^+^ T_CM_ were expressed at higher levels in NoTD than TD participants at baseline while CD27 in CD8^+^ T_CM_ appears to be at higher levels in TD than in NoTD participants (**Figure 8A**). We next assessed MEM scores and observed contrasting phenotypes in CD8^+^ T_CM_ between NoTD and TD participants. The phenotype of CD8^+^ T_CM_ in NoTD group was enriched with multiple effector markers (e.g., IL-17A, MIP1β, TNFα) while the TD group phenotype under-expressed effector markers (e.g., CXCR3 and PD-1) but was enriched for homing (CCR7) and activation (CD27) markers (**Figure 8A**). Following Ty21a vaccination, T-REX analysis similarly showed various regions of significant change (≥90%) containing clusters for NoTD and TD among other smaller clusters (**Figure 8B**). These NoTD and TD CD8^+^ T_CM_ clusters displayed distinct and contrasting levels of expression of activation, functional and homing markers as shown in the bubble plots (**Figure 8B**). For example, CXCR3, PD-1, MIP1β, IL-21 and GzB in CD8^+^ T_CM_ were expressed in higher levels in NoTD than TD participants following Ty21a vaccination while CCR7, CD27 and integrin α4β7 in CD8^+^ T_CM_ were increased in TD compared to NoTD participants (**Figure 8B**). MEM analysis showed that CD8^+^ T_CM_ in the NoTD group is enriched for multiple effector markers (e.g., MIP1β, TNFα, IL-21, PD-1, GzB, CXCR3) while the TD group under-expressed effector responses (e.g., IL-21, CXCR3, PD-1, MIP1β) but was enriched in homing (integrin α4β7 and CCR7) and activation (CD27) markers following Ty21a vaccination (**Figure 8B**).

Following *S*. Typhi challenge at D7, we observed two main regions of significant change (≥90%) in CD8^+^ T_CM_ containing clusters for NoTD and TD among other clusters (**Figure 8C**). The expression of markers again was different as NoTD and TD CD8^+^ T_EM_ clusters displayed distinct levels of activation, functional, and homing markers as shown in bubble plot (**Figure 8C**). For example, CD8^+^ T_CM_ from the TD participants showed a less activated phenotype with cytokines such as IL-17A and IFNγ being expressed at lower levels than in NoTD participants following wt *S*. Typhi challenge (**Figure 8C**).

MEM analysis showed that CD8^+^ T_CM_ in the NoTD group is enriched with multiple effector markers (e.g., MIP1β, CD27, CXCR5) while the TD group phenotype under-expressed effector responses (IL-17A) following wt *S*. Typhi challenge (**Figure 8C**). Finally, 28 days following challenge, we observed few pockets of >90% difference between TD and NoTD (**Figure 8D**). Interestingly, as shown in the bubble plots, the expression marker profile of TD participants is distinct from those of NoTD (**Figure 8D**). For example, TD CD8^+^ T_CM_ displayed higher levels of IL-17A, IL-21, IFNγ, and PD-1, than in NoTD participants post-challenge (D28) (**Figure 8D**). MEM analysis showed that CD8^+^ T_CM_ NoTD group is only enriched for the expression of CXCR3 while the TD participants display enrichment in the expression of CD27 (**Figure 8D**) following post-challenge at D28. Collectively, these phase-specific T-REX analyses delineate divergent immunophenotypic trajectories of CD8^+^ T_CM_ between TD and NoTD individuals.

### 3.9 CD8^+^T_EMRA_ unsupervised analysis and phenotype structure

We next used the UMAP reference map generated above and gated on CD45RA^+^ CD62L^-^ CD8^+^ T cells which represent CD45RA^+^ effector memory T cells (CD8^+^ T_EMRA_) to visualize and assess the distribution of activation, functional (e.g., cytokines), and homing markers (**Figures 9A–C**).

**Figure 9:**
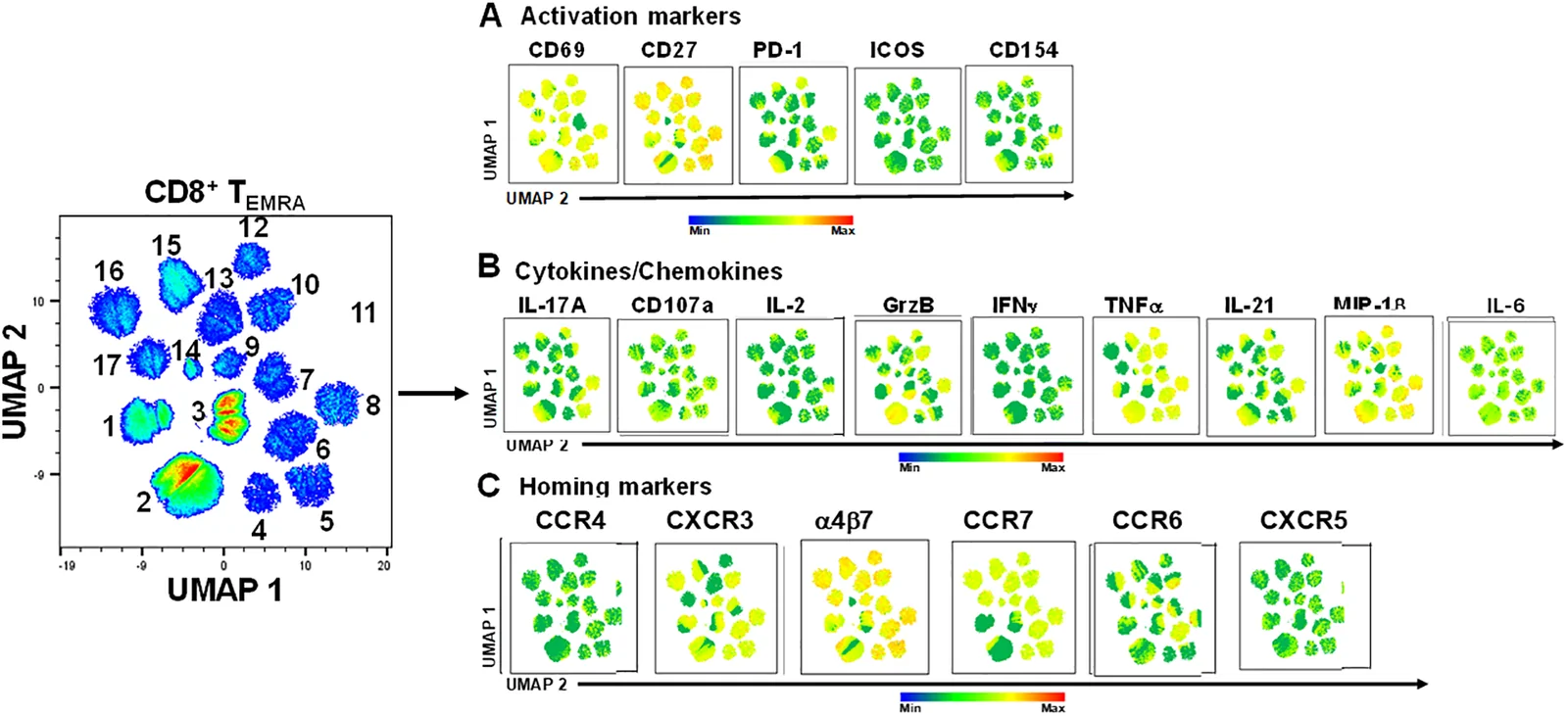
Unsupervised clustering and phenotype of TD and NoTD memory CD8^+^ T_EMRA_. The distribution of memory CD8^+^ T_EMRA_ subset within the 17 clusters were determined. **(A)** activation markers (CD69, CD27, PD-1, ICOS, CD154), **(B)** Cytokines and Chemokines (IL-17A, CD107a, IL-2, GranzymeB (GzB), IFNγ, TNFα, IL-21, MIP1β and IL-6), and **(C)** homing markers (CCR4, CXCR3, CD62L, integrin α4β7, CCR7, CCR6 and CXCR5) were evaluated in the 17 clusters. Scale bars indicate relative marker intensity.

CD8^+^ T_EMRA_ clusters displayed partially overlapping but distinct marker combinations, consistent with T_EMRA_ clusters suggestive of more terminally differentiated or cytotoxic programs (**Figures 9A–C**). For example, we observed that activation markers (CD69, CD27, PD-1, ICOS, CD154) were distributed in unique patterns across the 17 clusters on the UMAP (**Figure 9A**). Similar observations were made for cytokines/chemokines (e.g., IL-17A, CD107a, GzB, TNFα) (**Figure 9B**) and for homing markers particularly CXCR3, CCR6 and CCR7 (**Figure 9C**). These data suggested that CD8^+^T_EMRA_ clusters have unique combinations of markers that could be important in either Ty21a vaccination or protection from wt *S*. Typhi challenge.

### 3.10 *S*. Typhi-responsive CD8^+^ T_EMRA_ immune profile differs between TD and NoTD groups

Studies from our group have shown that *S.* Typhi-responsive CD8^+^ T_EMRA_ were induced following oral Ty21a vaccination albeit at a generally lower magnitude than the dominant CD8^+^ T_EM_ responses (Wahid et al., 2015). In CHIM studies, CD8^+^ T_EMRA_ were also detected within the post-exposure memory response after wild-type *S.* Typhi challenge, but they displayed more limited heterologous cross-reactivity than CD8^+^ T_EM_ (Rapaka et al., 2020). These data suggest that CD8^+^ T_EMRA_ play a measurable role in vaccine-elicited cellular immunity and participate in typhoid-responsive memory. However, the immune programming of CD8^+^ T_EMRA_ in TD and NoTD participants remains incompletely understood. Thus, we determined whether CD8^+^T_EMRA_ clusters frequencies differed between TD and NoTD participants following stimulation with *S*. Typhi- infected and uninfected targets.

Using ClusterExplorer, the frequencies of CD8^+^T_EMRA_ cluster 1-17 were assessed in TD and NoTD participants. We observed that there were various clusters that were present at higher frequencies in NoTD than in TD participants as shown by the yellow color intensity (**Supplementary Figure 8A**). For example, clusters 5, 12,14 and 17 were more frequent in NoTD than in TD participants (**Supplementary Figure 8A****).** However, these comparisons included all time points and stimulation conditions. Similarly, when CD8^+^ T_EMRA_ cluster frequencies were compared across stimulation conditions, some clusters (e.g., cluster 3, 5 and 17) were present at higher frequencies following stimulation with ST than with NI (**Supplementary Figure S8B**). However, these comparisons included all time points and participants. Thus, to interpret how *S*. Typhi-specific CD8^+^T_EMRA_ clusters vary between TD and NoTD over time, we deconvoluted the data as shown previously for CD8^+^ T_EM_ to evaluate the effects of Ty21a vaccination and wt *S*. Typhi challenge and determined net *S*. Typhi-specific CD8^+^ T_EMRA_ cluster responses. We then compared the frequencies of each *S*. Typhi-specific CD8^+^ T_EMRA_ cluster (Levine et al., 1987; Simanjuntak et al., 1991; Levine et al., 1999; Salerno-Goncalves et al., 2002; Salerno-Goncalves et al., 2004; Salerno-Goncalves et al., 2005; Salerno-Goncalves et al., 2010; Waddington et al., 2014; Klemm et al., 2018; Shakya et al., 2019; Jamilah et al., 2020; Patel et al., 2021; Qadri et al., 2021; Shakya et al., 2021; Sztein and Booth, 2022; Batool et al., 2024; Neuzil et al., 2024) in either TD or NoTD participants at each time point (**Figure 11**; **Supplementary Figure 9**). Next, we asked whether net *S.* Typhi-responsive CD8^+^ T_EMRA_ clusters differed by clinical outcome across the study period. ROC curves were used to determine statistically (AUC and p-value) between NoTD and TD participants. MEM was used to phenotype *S*. Typhi-specific CD8^+^ T_EMRA_ clusters.

Interestingly, out of the 17 clusters, we observed that there were three *S.* Typhi-specific CD8^+^ T_EMRA_ clusters (clusters 5, 6 and 15) that were associated with protection (**Figure 10A**). *S*. Typhi-specific Cluster 5 in NoTD group showed higher frequencies in NoTD (red line) than in TD (blue line) participants at most time points with significant differences at baseline (D-28) (**Figure 10A**). Using ROC, we observed significant (p<0.05) statistical differences between NoTD and TD group across all phases of the study (**Figure 10A**). Next, CD8^+^ T_EMRA_ cluster 5 was phenotype using MEM. The analysis showed that it is enriched for CD27, CCR7, MIP1β and IL-17A (**Figure 10A**). Similarly, the frequencies of *S*. Typhi-specific cluster 6 were observed to be higher in NoTD than TD participants at all time points in the study with significant differences (P<0.05) observed between the two groups using ROC (**Figure 10A**).

**Figure 10:**
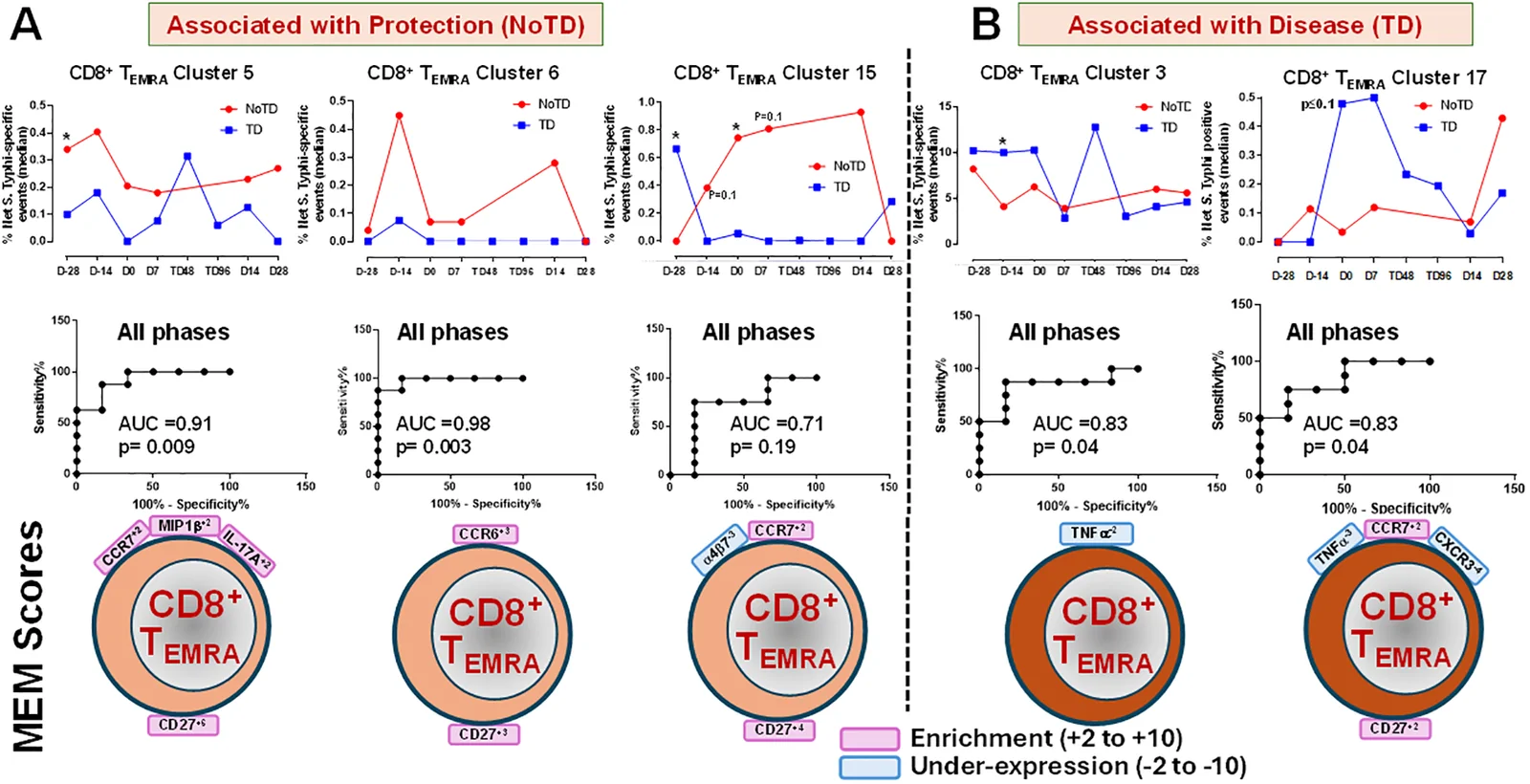
Unique *S*. Typhi-specific CD8^+^ T_EMRA_ clusters are associated with protection (NoTD) and disease (TD) outcomes. Net *S*. Typhi responses of CD8^+^ T_EMRA_ clusters were determined in TD and NoTD PBMC samples at all time points (D-28 to D28). *S*. Typhi-specific CD8^+^ T_EMRA_ associated with **(A)** Protection and **(B)** Susceptibility (Disease) were identified (NoTD (Red); TD (Blue)). Receiver operating characteristic (ROC) curves summarize discriminatory performance (area under the curve [AUC] and p-value) between NoTD and TD groups during the phases. Marker Enrichment Modeling (MEM) signatures for each cluster are shown highlighting enriched (MEM + 2 to +10; pink) and under-expressed (MEM −2 to −10; blue) features. Significant differences between TD and NoTD participants at each time points are represented by * p<0.05. P-values are indicated for trends. Values are shown as median % of events.

The phenotype of CD8^+^ T_EMRA_ cluster 6 as determined by MEM displayed an enrichment in CD27 and CCR6 (**Figure 10A**). Subsequently, *S*. Typhi-specific Cluster 15 in CD8^+^ T_EMRA_ showed higher frequencies in NoTD (red line) than in TD (blue line) participants at most time points with significant differences at baseline (D-28) and following Ty21a vaccination (D0) (**Figure 10A**). Using ROC, we observed no significant (p<0.05) statistical differences between NoTD and TD group across all phases of the study (**Figure 10A**). CD8^+^ T_EMRA_ cluster 15 was phenotype using MEM and we observed that it is enriched for CD27 and CCR7 but a decrease in integrin α4β7 (**Figure 10A**).

In contrast, out of the 17 clusters, we also observed that CD8^+^ T_EMRA_ cluster 3 and 17 were associated with susceptibility to typhoid disease (**Figure 10B**). The frequencies of *S*. Typhi-specific cluster 3 were observed to be higher in frequencies in TD than in NoTD participants at baseline and following Ty21a vaccination (D-14 and D0) with significant differences between the two groups at D-14 after vaccination (**Figure 10B**). ROC showed that there were significant differences (p<0.05) between the two groups (**Figure 10B**). The phenotype of CD8^+^ T_EMRA_ cluster 3 as determined by MEM displayed under-expression of TNFα (**Figure 10B**). *S*. Typhi-specific cluster 17 similarly displayed higher level in CD8^+^ T_EMRA_ frequencies in TD than NoTD participants following Ty21a vaccination (D0) and following *S.* Typhi challenge (D7) (**Figure 10B**). ROC showed that there were significant differences (p<0.05) between the two groups across all phases (**Figure 10B**). The phenotype of CD8^+^ T_EMRA_ cluster 17 as determined by MEM displayed an under-expression of TNFα and CXCR3 but an enrichment in CD27 and CCR7 (**Figure 10B**). Collectively, these findings identify net *S.* Typhi-specific CD8^+^ T_EMRA_ clusters which are either associated with protection or susceptibility to typhoid disease. These clusters have unique phenotypic signatures and differential kinetics between NoTD and TD participants. Together, these findings suggest that outcome discrimination is distributed across multiple memory compartments, with T_EM_ and T_EMRA_ subsets providing complementary insights into effector and terminal effector programs following vaccination and challenge.

### 3.11 T-REX analysis uncovers phase-specific divergence of TD and NoTD CD8^+^ T_EMRA_ immune profiles

Equal numbers of CD8^+^ T_EMRA_ events from TD and NoTD participants at each time point were concatenated and subjected to T-REX analysis as described above for CD8^+^ T_EM_. At baseline (D-28), T-REX revealed two regions of significant change (≥90%) containing CD8^+^ T_EMRA_ clusters for NoTD (red) and TD (blue) participants in addition to other clusters (**Figure 11A**). These NoTD and TD CD8^+^ T_EMRA_ clusters displayed distinct and contrasting levels of expression of activation, functional and homing markers as shown in the bubble plots (**Figure 11A**). Remarkably, CD8^+^ T_EMRA_ from NoTD participants expressed higher levels of CXCR3, CD154, CD107a and IL-17A than TD participants at baseline while GzB and integrin α4β7 markers in CD8^+^ T_EMRA_ seem to be at higher levels in TD than in NoTD participants (**Figure 11A**). MEM analysis demonstrated different phenotypes in CD8^+^ T_EMRA_ between the NoTD and TD groups (**Figure 11A**). The phenotype of CD8^+^ T_EMRA_ NoTD group was enriched with multiple effector markers (e.g., IL-17A, MIP1β, TNFα) while the TD group phenotype under-expressed the CXCR5 marker but was enriched for homing (integrin α4β7) and functional (GzB) markers (**Figure 11A**).

**Figure 11:**
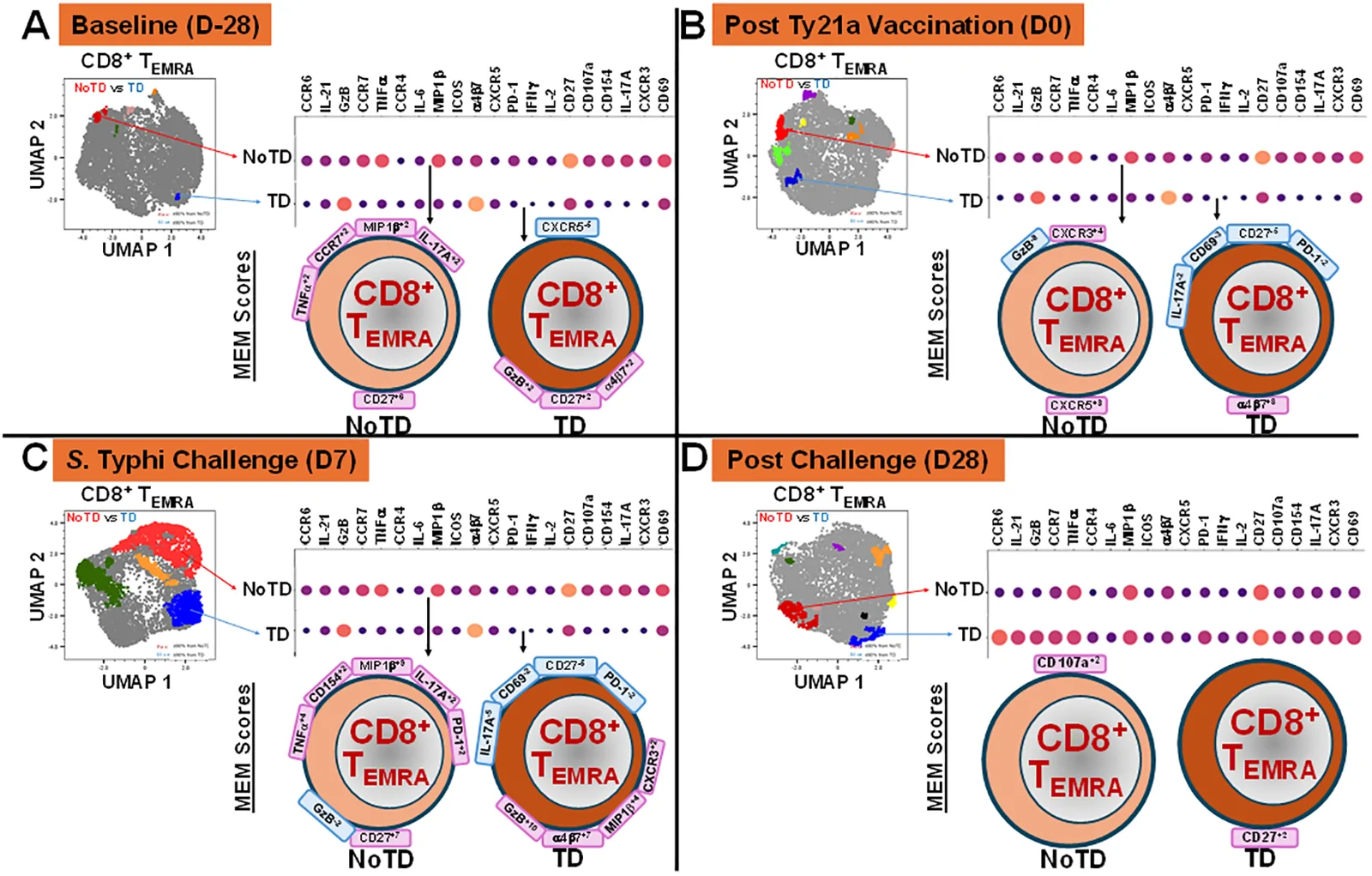
Diverse phase-specific TD vs NoTD CD8^+^ T_EMRA_ signature program using T-REX analysis. Tracking Responders Expanding (T-REX) analysis was performed at each time point representing the different phases of the study to compare NoTD vs TD at **(A)** baseline (D-28), **(B)** post Ty21a vaccination (D0), **(C)** post *S*. Typhi challenge (D7), and **(D)** day 28 post-challenge (D28) in CD8^+^ T_EMRA_ stimulated with *S*. Typhi-infected targets. Regions/clusters showing >90% difference were identified as unique to TD (blue) and NoTD (red). Dot/bubble summaries depict relative expression of measured markers in NoTD vs TD for the highlighted regions (color intensity corresponds to scaled marker level). MEM score thresholds shown in legend.

Following Ty21a vaccination, T-REX analysis similarly showed various regions of significant change (≥90%) containing clusters for NoTD and TD in addition to smaller clusters (**Figure 11B**). These regions showed distinct patterns of activation, functional, and homing marker expression between NoTD and TD groups, as shown in the bubble plots (**Figure 11B**).

For example, CXCR3, CD107a, CD27, and TNFα in CD8^+^ T_EMRA_ were expressed at higher levels in NoTD than in TD participants following Ty21a vaccination while GzB and integrin α4β7 in CD8^+^ T_EMRA_ were increased in TD compared to NoTD participants (**Figure 11B**). MEM analysis showed that CD8^+^ T_EMRA_ phenotype in the NoTD group was enriched with CXCR3 and CXCR5 and under-expressed GzB while the TD group phenotype under-expressed effector responses (CD69, IL-17A, CD27, and PD-1) but was enriched in homing (integrin α4β7) markers following Ty21a vaccination (**Figure 11B**). Following *S*. Typhi challenge at D7, we observed two main regions of significant change (≥90%) in CD8^+^ T_EMRA_ containing clusters for NoTD and TD participants in addition to other clusters (**Figure 11C**). The expression of markers again was different as NoTD and TD CD8^+^ T_EMRA_ clusters displayed distinct levels of activation, functional, and homing markers as shown in the bubble plots (**Figure 11C**).

For example, CD8^+^ T_EMRA_ from the TD group showed a less activated phenotype with IL-17A, CD154, CD107a, IL-2, IFNγ, PD-1, and TNFα being expressed at lower levels than in NoTD participants following wt *S*. Typhi challenge (**Figure 11C**). The phenotype determined by MEM score showed that CD8^+^ T_EMRA_ in the NoTD group was enriched with multiple effector markers (e.g., MIP1β, CD27, IL-17A, TNFα, CD154 and PD1) while the TD group phenotype under-expressed effector responses (IL-17A, PD-1, CD27 and CD69) but was enriched in GzB, integrin α4β7, MIP1β and CXCR3 following wt S. Typhi challenge (**Figure 11C**).

Finally, 28 days following challenge, we observed few pockets of >90% differences between TD and NoTD (**Figure 11D**). Interestingly, as shown in bubble plot, the expression marker profile of TD participants is distinct from those of NoTD (**Figure 11D**). For example, TD CD8^+^ T_EMRA_ displayed higher levels of CCR6, IL-17A, IL-21, PD-1, GzB, CXCR3, and CD69 than in NoTD participants post-challenge (D28) (**Figure 11D**).

MEM analysis showed that CD8^+^ T_EMRA_ in the NoTD group is downregulated as it is only enriched in the expression of CD107a while the TD group displayed enrichment in CD27 (**Figure 11D**) post-challenge at D28. Collectively, these phase-specific T-REX analyses highlight divergent CD8^+^ T_EMRA_ immunophenotypic programs in TD and NoTD participants across the study timeline.

### 3.12 CD8^+^ T subsets as predictors of clinical outcome by feature-ranking analysis

To determine the set of features contributing to clinical outcome discrimination from each CD8^+^ T_M_ subset (T_EM_, T_CM_, T_EMRA_), we applied feature-ranking analysis to quantify the predictive value of each immune feature from supervised analysis of T_M_ subsets. This approach highlighted a limited number of markers from each CD8^+^ T_M_ subset that contributed to discrimination between TD and NoTD outcome. We used Gini importance scores to quantify the predictive value of each immune feature. High Gini scores indicate strong discriminators between clinical outcomes, helping us prioritize markers linked to protection or susceptibility. The results of the analysis of features that show significantly differential marker expression between TD vs NoTD (Wilcoxon rank-sum tests p< 0.1) are shown in **Figure 12**. The blue bars represent specific features associated with development of typhoid disease (TD) while the red bars represent features associated with protection. Following feature ranking analysis, we identified the top features associated with NoTD across the study phases for each CD8^+^ T memory subset. For T_EM_, CXCR5 following Ty21a immunization and IL-17A following wt *S*. Typhi challenge were identified as predictive features for protection (**Figure 12**). In contrast, the top features in CD8^+^ T_EM_ associated with susceptibility to typhoid disease were integrin α4β7, CD27 in the baseline and immunization phases while CCR6, CD27 and GzB were the top features following wt *S.* Typhi challenge (**Figure 12**).

**Figure 12:**
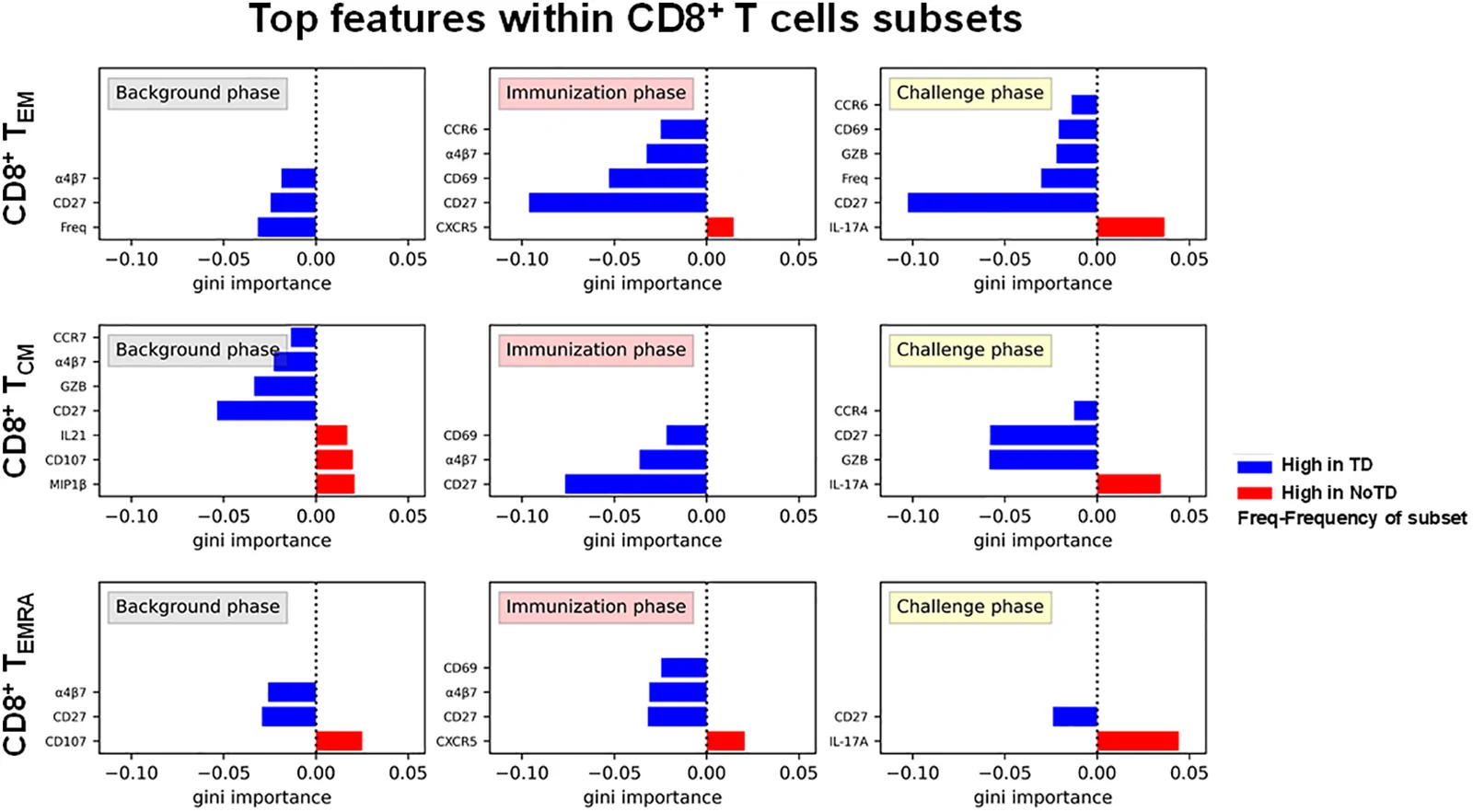
Identification of CD8^+^ T_EM_, T_CM_, T_EMRA_ subset-specific predictors of clinical outcome by machine learning (Gini performance). Gini importance was used by trained models to quantify the predictive value of each immune feature from supervised analysis of T_M_ subsets. We evaluated the Gini importance score in CD8^+^ T_EM_, T_CM_ and T_EMRA_ across each study phase: baseline, immunization, and challenge. The blue bars represent specific features associated with development of typhoid disease (TD) while the red bars represent features that are associated with protection.

For CD8^+^ T_CM_, we observed that in background phase the top predictive features for protection were IL-21, CD107a and MIP1β while in the challenge phase, the predictive feature for protection was IL-17A, as was also the case for CD8^+^ T_EM_ (**Figure 12**). In contrast, the top predictive features for susceptibility to TD in CD8^+^ T_CM_ were CD27, GzB and integrin α4β7 in the background and immunization phase while in the challenge phase, the top features for CD8^+^ T_CM_ was CD27 and GzB (**Figure 12**).

Finally, for CD8^+^ T_EMRA_, we noted that the top features for predicting protection were CD107 (background), CXCR5 (immunization) and IL-17A (challenge) (**Figure 12**). In contrast, the top predicting features for susceptibility to TD in CD8^+^ T_EMRA_ were CD27 and integrin α4β7 in the background and immunization phases and CD27 in the challenge phase (**Figure 12**). These analyses provided an important bridge between subset-level discovery and biologically interpretable marker programs. Altogether these complementary analytic methods converge on distinct immune response signatures following Ty21a vaccination and wt *S*. Typhi challenge in all three CD8^+^ T_M_ subsets.

### 3.13 Conventional gating of CD8^+^ T_CM_ and T_EMRA_ confirms key dynamics identified by unsupervised analysis

Next, we validated our unsupervised findings using conventional supervised gating of ex-vivo CD8^+^ T_CM_ and T_EMRA_ in PBMC obtained from NoTD and TD participants across all study time points. Remarkably, consistent with the unsupervised analysis, the signature markers of CD8^+^ T_CM_ associated with protection such as CXCR3 (baseline and Ty21a vaccination) (**Figure 13A**) and IL-17A (D7 after *S.* Typhi challenge) (**Figure 13B**) are higher in NoTD than in TD participants. Furthermore, when we gated on CD8^+^ T_CM_ cells co-expressing a combination of protection-associated markers (e.g., CD27^+^ IL-17A^+^ TNFα^+^), we found that this subset was more frequent in NoTD than in TD participants across all time points, with a significant difference observed on Day 7 following *S.* Typhi challenge (**Figure 13C**). We next performed ROC analysis and showed that CD8^+^ T_CM_ expressing CXCR3 and the co-expression of CD27, IL-17A and TNFα displayed significant differences (p=0.002 and p=0.009 respectively) in all phases between NoTD and TD group (**Supplementary Figures 10A–C**). Similar findings were made from CD8^+^ T_EMRA_ subset. Markers of CD8^+^ T_EMRA_ such as CXCR3 (baseline and Ty21a vaccination) (**Figure 13D**) and IL-17A (significant (p<0.05); D7 after S. Typhi challenge) (**Figure 13E**) were expressed higher in NoTD than in TD participants. Combinations of protection-associated markers, such as in the CD8^+^ T_EMRA_ co-expressing CD27, IL-17A, and TNFα, were consistently more abundant in NoTD than TD participants across all time points (**Figure 13F**). ROC analysis showed that CD8^+^ T_EMRA_ expressing CXCR3, IL-17A and the co-expression of CD27, IL-17A and TNFα displayed significant differences (p=0.004, p=0.003 and p=0.04 respectively) in all phases between NoTD and TD groups (**Supplementary Figures 10D, E**).

**Figure 13:**
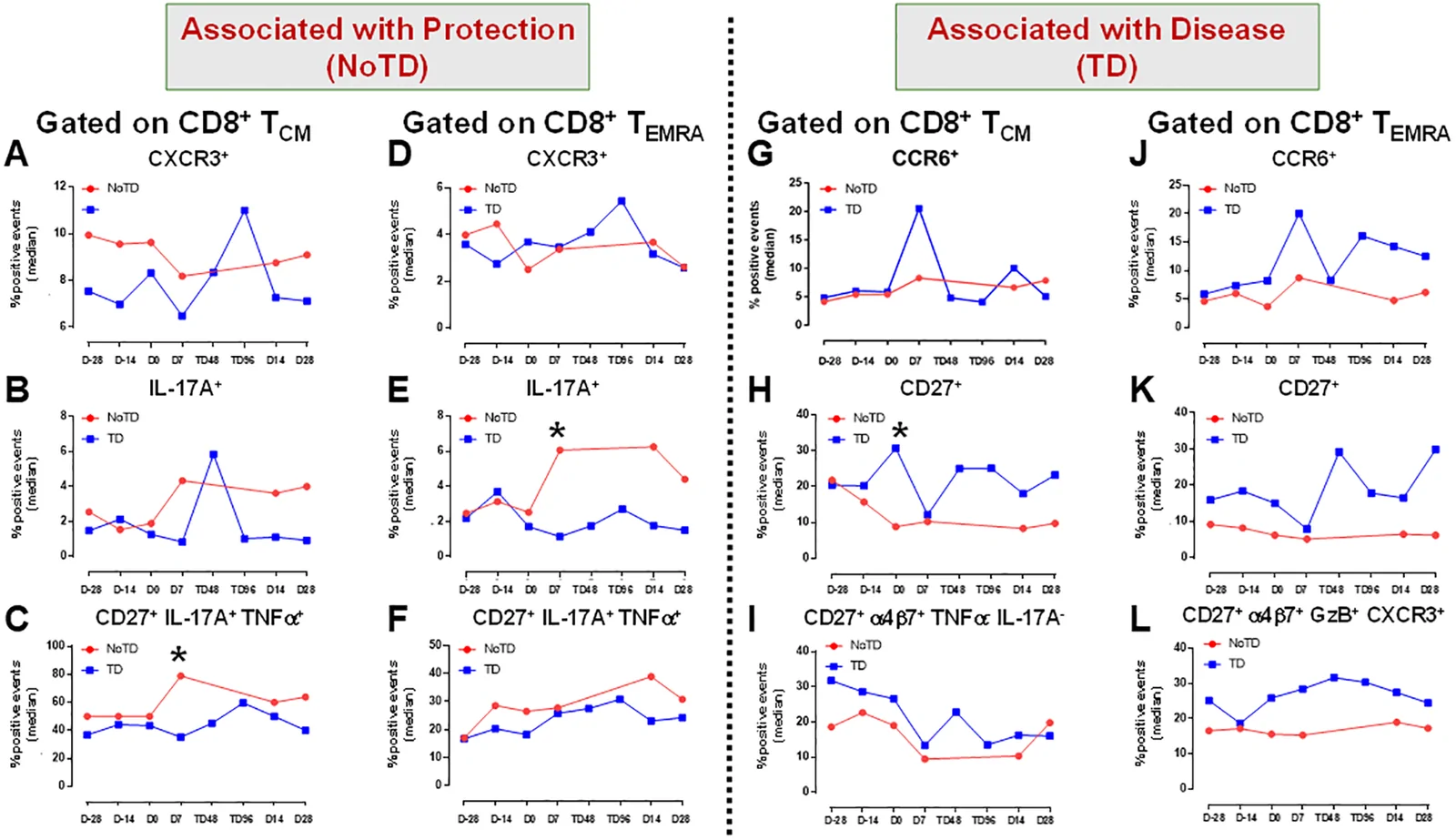
Validation of clinical outcome-associated CD8^+^ T_CM_ and T_EMRA_ signature markers by manual gating. Using CD8^+^ T_CM_ and T_EMRA_ subsets obtained ex-vivo PBMC from participants in both TD and NoTD groups at all time points (D-28 to D28), conventional supervised analysis was performed to determine signature markers associated with TD and NoTD. CD8^+^ T_CM_ and T_EMRA_ markers associated with protection (NoTD), **(A, D)** CXCR3, **(B, E)** IL-17A, **(C, F)** CD27^+^ IL-17A^+^ TNFα<συπ>+</sup> and those associated with typhoid disease, **(G, J)** CCR6, **(H, K)** CD27 respectively, **(I)** T_CM_: CD27^+^ integrin α4β7^+^ TNFα^-^ IL-17A^-^ and **(L)** T_EMRA_: CD27^+^ integrin α4β7^+^ GzB^+^ CXCR3^+^ were determined and compared between TD and NoTD across all time points in the study. Significant differences between TD and NoTD volunteers at each time points are represented by *p<0.05.

In contrast, CD8^+^ T_CM_ and T_EMRA_ signature markers associated with susceptibility were found to be significantly higher in the TD than in the NoTD group (**Figures 13G–L**). Consistent with the unsupervised analysis, the signature markers of CD8^+^ T_CM_ associated with susceptibility to disease (TD) such as CCR6 (D7 after *S*. Typhi challenge) (**Figure 13G**) and CD27 (Significant (p<0.05) after Ty21a vaccination (D0)) (**Figure 13H**) are higher in TD than in NoTD participants.

Furthermore, when we gated on CD8^+^ T_CM_ cells co-expressing a combination of susceptibility-associated markers (e.g., CD27^+^ integrin α4β7^+^ IL-17A^-^ TNFα^-^), we found that this subset was consistently more frequent in TD than in NoTD participants across all time points (**Figure 13I**). ROC analysis showed that CD8^+^ T_CM_ expressing CD27 displayed significant differences (p=0.005) in all phases between NoTD and TD groups (**Supplementary Figure 10H**).

Similar findings were made in the CD8^+^ T_EMRA_ subset. Markers such as CCR6 (Ty21a vaccination, D7 after *S.* Typhi challenge) (**Figure 13J**) and CD27 (Baseline, Ty21a vaccination) (**Figure 13K**) were expressed at higher levels in CD8^+^ T_EMRA_ of TD than in NoTD participants. In addition, CD8^+^ T_EMRA_ co-expressing susceptibility-associated markers CD27, integrin α4β7, GzB and CXCR3 were consistently more abundant in TD than NoTD participants across all time points (**Figure 13L**). ROC analysis showed that CD8^+^ T_EMRA_ expressing CCR6, CD27 and co-expression of CD27, integrin α4β7, GzB and CXCR3 displayed significant differences (p<0.005) between NoTD and TD groups (**Supplementary Figures 10J–L**). Thus, this cross-validation strengthens the evidence that distinct CD8^+^ T_CM_ and T_EMRA_ functional programs are associated with susceptibility or protection from typhoid disease.

## 4 Discussion

Protective immunity to *S*. Typhi is complex, multifactorial and remains incompletely defined, particularly with respect to the cellular correlates of protection. CHIM and vaccination studies have demonstrated that *S*. Typhi-responsive CD8^+^ T cells are one of the essential components of host immunity against typhoid, particularly with findings that focused on multifunctional CD8^+^ T_EM_ and on gut-homing T-cells at baseline (Fresnay et al., 2016; Fresnay et al., 2017). However, we still lack a high-dimensional understanding of the memory CD8^+^ T cell subsets programs that determine protection against typhoid disease. Thus, identifying the immunological profiles of CD8^+^ T cells in *S*. Typhi infection and Ty21a vaccination as they relate to protection from disease is necessary to better define the immunological correlates of protection.

Here, we investigated in depth the role of memory CD8^+^ T and its subsets in participants who developed typhoid disease (TD) or not (NoTD) following Ty21a vaccination and *S*. Typhi challenge. We observed that protection from typhoid disease is not merely associated with the presence of CD8^+^ T memory responses, but with divergent CD8^+^ T immunophenotypic programs which are distinct and phase-specific and distributed across multiple CD8^+^ memory compartments (T_EM_, T_CM_, and T_EMRA_). In volunteers who did not develop disease (NoTD group), we found that CD8^+^ T_EM_ cells at baseline are MF and Ty21a vaccination enhances the development of Tc17, Tc1 and T_FH_ which become dominant in the challenge phase. These findings suggest that CD8^+^ T_EM_ cells in NoTD participants exhibit a multifunctional inflammatory program, including increased IL-17A expression, that may support mucosal barrier defense and neutrophil-associated antimicrobial responses and thereby contributing to improved control of *S.* Typhi and no progression to typhoid disease (**Figure 14**). In contrast, in participants who develop disease (TD), we observed that CD8^+^ T cells are limited in cytokine diversity at baseline and following Ty21a vaccination. These CD8^+^ T_EM_ have a modest increase in homing markers such as CCR6, integrin α4β7, CD27 and GzB with limited diversity of effector molecules (**Figure 14**). This CD8^+^ T cell profile became dominant during the challenge phase leading to a restricted effector profile by integrin α4β7^+^ CD8^+^ T_EM_ cells that are nevertheless likely to home to the gut. This pattern is consistent with a gut-homing and cytotoxicity-biased program and may reflect redistribution of these cells from the circulation; however, it should be interpreted as an immune signature associated with typhoid disease (**Figure 14**). Direct assessment of intestinal tissue responses, cellular migration, infected-target-cell killing, and bacterial control will be required to determine the functional consequences of this phenotype. Thus, our study revealed that distinct *S*. Typhi-specific CD8^+^ T_EM_ profiles across baseline, Ty21a vaccination, and challenge phases are associated with clinical outcome. In addition, using several complementary analytical methods, we have found core markers associated with protection for CD8^+^ T_EM_ (NoTD- CXCR5, IL-17A, CXCR3, TNFα, PD-1, MIP1β, CD27 and IL-21) and with susceptibility (TD- CCR6, CD27, integrin α4β7 and GzB). Notably, we also found that these observations can be extended to other memory CD8^+^ T cell compartments including T_CM_ and T_EMRA_ (**Figure 15**). Furthermore, translational approaches in other fields such as immuno-oncology have also demonstrated that multidimensional immune signatures, including T-cell–inflamed gene-expression profiles, can predict clinical response and be incorporated into biomarker-directed clinical trials, underscoring the broader value of linking high-dimensional immune profiles with clinical outcomes (Ayers et al., 2017; Gutierrez et al., 2023; Narote et al., 2025; Iamukova and Alferova, 2026). Taken together, these data contribute major novel information of the role of memory CD8^+^ T cells and its subsets following oral Ty21a vaccination and *S*. Typhi infection as they relate to development of disease following oral exposure to wt *S.* Typhi in humans.

**Figure 14:**
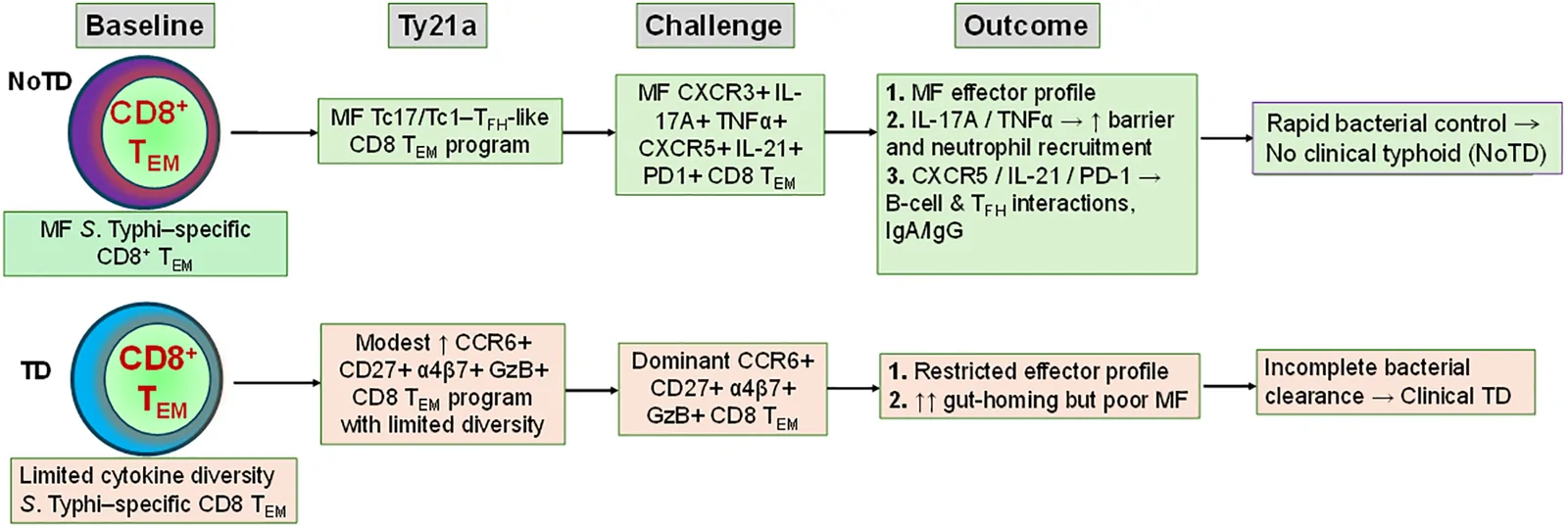
Model of divergent CD8^+^ T_EM_ program with typhoid disease outcome. This model summarizes the CD8^+^ T_EM_ signature profiles identified across baseline, Ty21a immunization, and wild-type *S. Typhi* challenge. In protected participants (NoTD), CD8^+^ T cells at baseline are associated with multifunctional (MF) profile and Ty21a vaccination enhances the development of T cytotoxic 17 (Tc17), T cytotoxic 1 (Tc1) and T follicular/helper cells (T_FH_) signature profiles which would become dominant in the challenge phase. The predictive outcome is that CD8^+^ T cells would become effective MF effectors with potentially higherIL-17A barrier function and neutrophil recruitment which will allow for rapid clearance of *S*. Typhi leading to no typhoid disease (NoTD). Such a program may support coordinated mucosal host-defense responses and improved control of *S. Typhi*, In contrast, TD CD8^+^ T cells are associated with limited cytokine diversity and following Ty21a, they displayed a modest increase in signature markers which would become dominant during the challenge phase. This potentially will result in a restricted, non-effective effector profile accompanied by gut homing potential leading to incomplete bacterial clearance and typhoid disease.

**Figure 15:**
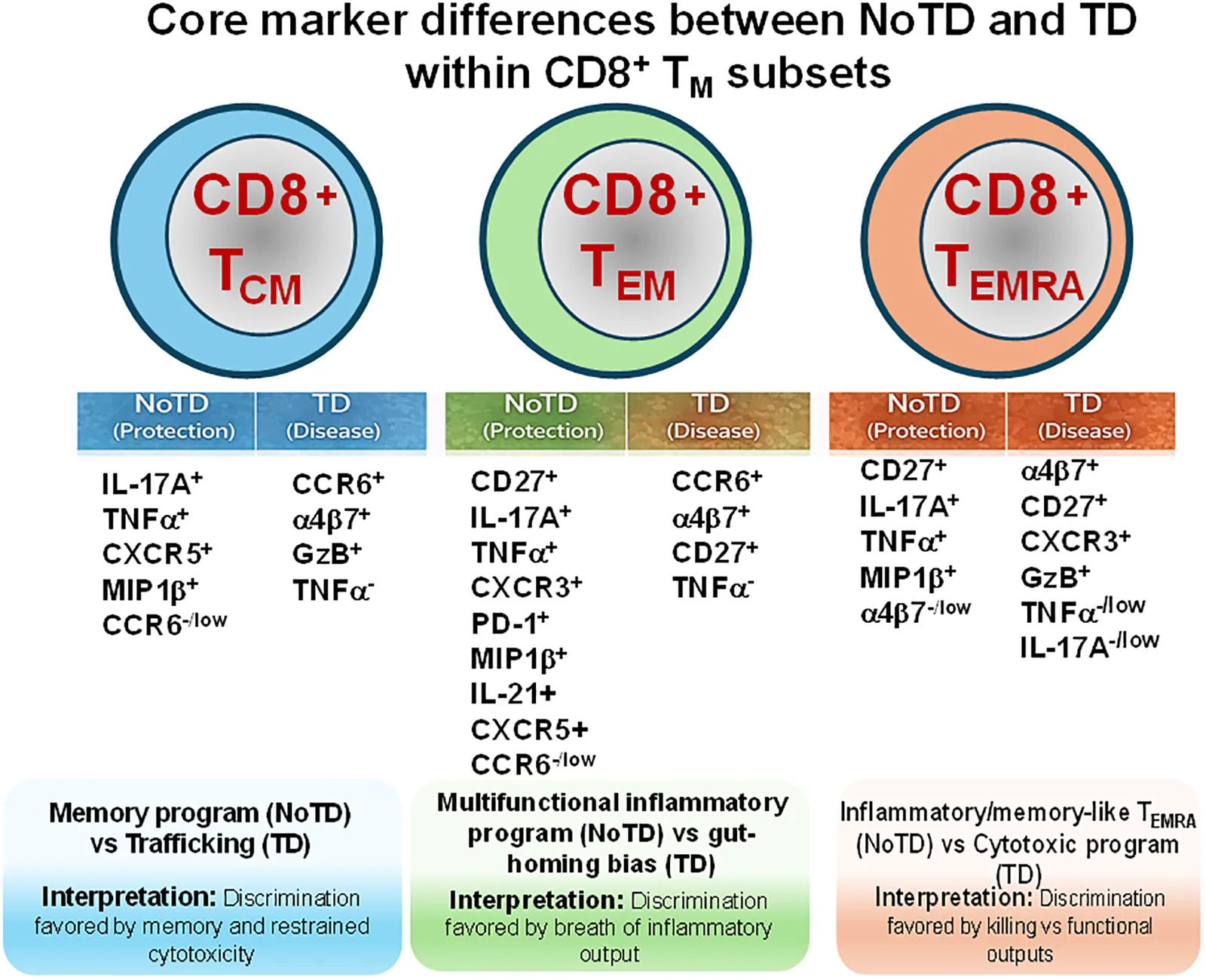
Comparison of typhoid related CD8^+^ T memory subsets immune signature associated with disease outcome. This summary model integrates the dominant outcome-associated immune signatures identified within CD8^+^ T_CM_, T_EM_, and T_EMRA_ subsets across the Ty21a vaccination and wild-type *S. Typhi* challenge timeline. This diagram summarizes the dominant outcome-associated signatures identified in CD8^+^ T_CM_, T_EM_, and T_EMRA_ subsets across the timeline of the vaccination/CHIM study. Across these subsets, protection (NoTD) was associated with immune programs consistent with memory-associated, multifunctional inflammatory, and inflammatory/memory-like features, whereas TD participants displayed associated profiles enriched for trafficking, gut-homing bias, or cytotoxic programs. Together, these findings highlight qualitatively distinct, subset-specific CD8^+^ T-cell memory programs associated with protection from versus development of typhoid disease.

The divergent CD8^+^ T memory trajectories observed in our study are consistent with prior CHIM studies. Our group have shown that higher baseline multifunctional *S*. Typhi-specific CD8^+^ T responses were associated with protection and delayed diagnosis in a low-dose typhoid (*S*. Typhi- 1-5 x 10^3^ cfu) CHIM, and participants who developed typhoid disease showed a post-challenge decrease in circulating gut-homing CD8^+^ T_EM_ consistent with a different CD8^+^ T cell memory trajectory in protected versus diseased individuals (Fresnay et al., 2016). Similarly, in a high-dose typhoid (*S*. Typhi- 1-5 x 10^4^ cfu) CHIM, Fresnay et al., 2017 have reported again an outcome-linked divergence (Fresnay et al., 2017). They found that higher baseline *S*. Typhi-responsive CD8^+^ T cell memory responses were associated with greater risk of meeting diagnostic criteria, but among those who developed typhoid, stronger baseline responses were associated with delayed diagnosis (Fresnay et al., 2017). Our data presented herein are consistent with these observations showing that baseline *S*. Typhi-responsive CD8^+^ T memory responses are associated with subsequent typhoid outcome. These data support that pre-existing cellular immune states are associated with susceptibility or protection from disease. Outcome-linked divergence of CD8^+^ T memory programs is not unique to typhoid disease. Similar patterns have been reported in human influenza, HIV, and dengue, where protection or non-progression is associated with distinct pre-existing or evolving CD8 memory states rather than the magnitude of CD8^+^ T cell responses only (Northfield et al., 2007; Sridhar et al., 2013; Weiskopf et al., 2013; Ioannidis et al., 2023). For example, individuals who developed less severe pandemic H1N1 illnesses had higher frequencies of pre-existing CD8^+^ T cell responses to conserved epitopes (Sridhar et al., 2013). In addition, CD8^+^ CD45RA^+^ CCR7^−^ late-effector phenotype inversely correlated with symptom score in influenza (Sridhar et al., 2013). Furthermore, it was reported that uncomplicated secondary dengue was associated with expansion of ICOS^+^ CD8^+^ T_EM_ while in patients that progress to severe dengue, this response was largely absent (Ioannidis et al., 2023). Another study has reported that higher-magnitude, more multifunctional dengue-specific CD8^+^ T cell responses which were associated with HLA alleles linked to decreased susceptibility to severe disease (Weiskopf et al., 2013). Altogether, these studies provide supporting evidence for outcome-linked divergence in CD8^+^ T cell memory quality in infectious diseases.

One of the major implications of our findings is that protection appears to be associated with a multifunctional CD8^+^ T cell memory program rather than with any single marker in isolation. Across all of our unsupervised clustering, T-REX analyses, and conventional supervised-gating validation, we observed that protected (NoTD) participants were enriched for CD8^+^ T memory states expressing combinations of CXCR3, IL-17A, TNFα, MIP-1β, IL-21, PD-1, CD107a, and granzyme B, while participants who developed disease (TD) displayed CD8^+^ T memory states enriched for CCR6 and integrin α4β7 together with reduced expression of multiple effector-associated markers. Interestingly, we have previously reported that oral immunization induces multifunctional *S*. Typhi-responsive CD8^+^ T cells capable of producing combinations of IFN-γ, TNF-α, MIP-1β, and CD107a, as well as heterogeneous IL-17A-producing CD8^+^ T-cell populations following Ty21a vaccination (McArthur and Sztein, 2012; Wahid et al., 2015). Similarly, in the CHIM studies by Fresnay et al., multifunctional CD8^+^ T cells were associated with clinical outcome and the authors suggested that when exposed to a higher infectious dose, the quality and context of multifunctional CD8 responses may matter more than magnitude alone (Fresnay et al., 2017). We have also shown that Ty21a vaccination elicits multifunctional *S*. Typhi-responsive CD8^+^ memory T-cell responses in the human terminal ileum, supporting a role for mucosal CD8^+^ T cell immunity at the site of pathogen entry (Booth et al., 2019c). These published findings support the view that coordinated multifunctionality, rather than any single cytokine or activation marker in isolation, is a defining feature of protective typhoid-responsive cellular immunity.

It is interesting to note the importance of tissue-trafficking programs in shaping typhoid clinical disease outcome. We found that in protected participants (NoTD), the clusters associated with protection were consistently enriched for CXCR5 (at least in CD8^+^ T_EM_ and T_CM_) and other inflammatory effector functions. Whereas susceptibility-associated clusters in participants who developed disease (TD) were generally characterized by CCR6 (at least in CD8^+^ T_EM_ and T_CM_), integrin α4β7 expression, and under-expression of TNFα. We and others have shown that mucosal and systemic CD8^+^ T cells elicited following Ty21a vaccination have gut and lymph nodes homing potential (Lundin et al., 2002; Wahid et al., 2008; Wahid et al., 2015). Moreover, we reported in CHIM studies that development of typhoid disease was associated with decreases in circulating *S*. Typhi-responsive CD8^+^ T_EM_ cells with gut-homing potential, suggesting redistribution to sites of infection (Fresnay et al., 2016). Together, these observations suggest that the balance between inflammatory effector competence and intestinal trafficking may be some of the key determinants of whether CD8^+^ T cell memory responses are associated with protection or susceptibility.

Our group has also shown that Ty21a vaccination elicits *S*. Typhi-responsive CD8^+^ T memory responses across T_CM_, T_EM_, and T_EMRA_ compartments. Multifunctional responses were predominantly observed in T_EM_ and, to a lesser extent, in T_EMRA_, with lower-magnitude responses detected in T_CM_ (Wahid et al., 2015; Booth et al., 2017). In CHIM studies, the strongest direct evidence implicated multifunctional CD8^+^ T_EM_, whilst T_CM_ and T_EMRA_ were not investigated thoroughly. In this CHIM study, we studied in considerable detail the involvement of CD8^+^ T_CM_ and T_EMRA_ in the development of disease. Our data indicated that both CD8^+^ T_CM_ and T_EMRA_ subsets also contain protection- and susceptibility-associated states which are likely to be biologically important. The identification of CXCR5-, TNFα, MIP1β, and IL-17A-enriched T_CM_ and T_EMRA_ populations in protected participants (NoTD) together with GzB/α4β7-biased and cytokine under-expression (e.g., TNFα<συπ>-</sup>) susceptibility-associated counterparts in participants who develop typhoid disease (TD), suggests that outcome-relevant immune programming is distributed across the broader CD8^+^ T memory landscape rather than confined to a single subset. This has not been observed at this resolution in previous typhoid studies. However, this has been reported in other human studies. For example, in HIV, preserved multifunctional CD8 T_CM_ responses are associated with viral control, whereas in dengue virus infection, activated multifunctional CD8 T_EM_/T_EMRA_ populations have been linked to protective immunity (Ndhlovu et al., 2012). In addition, pre-existing CD8 memory types in influenza correlate with reduced disease severity and viral burden (Sridhar et al., 2013; Paterson et al., 2021). Conversely, pro-inflammatory CD8 T_EMRA_ expansions have also been associated with post-acute COVID-19 sequelae, indicating that the biologic significance of T_EMRA_ depends on timing and context (Paniskaki et al., 2023). These studies support our contention that typhoid outcome is not simply linked by the magnitude of the CD8^+^ T responses, but by the composition, functionality, and temporal deployment of distinct CD8^+^ T cell subsets. Overall, these findings suggest that *S.* Typhi-specific CD8^+^ T_CM_, T_EM_, and T_EMRA_ responses represent interconnected memory programs, with T_CM_ reflecting immune readiness that may shape downstream T_EM_ responses, while T_EMRA_ provides complementary insight into terminal effector differentiation following Ty21a vaccination and *S.* Typhi challenge. Future studies integrating transcription-factor profiling, single-cell transcriptomic and epigenetic analyses, T-cell receptor clonotype tracking, functional characterization and independent cohort validation will be required to define the mechanisms responsible for the generation, maintenance, and clinical relevance of these outcome-associated CD8^+^ T-cell memory programs.

In this study there are several limitations that we need to acknowledge. In particular, the number of participants (e.g., n=8 for TD and n=8 for NoTD) was a relatively small cohort size, which might carry the risk of type II errors and overfitting, a possibility in exploratory studies in humans such as those involving high-dimensional profiling. Nevertheless, small, intensively characterized cohorts have been used in other high-dimensional immunological studies when specimens are rare or obtained from unique clinical studies, particularly those attempting to explore the association of immunological parameters with a clinical outcome. For example, Kramer et al. applied CyTOF, T-REX, and MEM to longitudinal samples from ten vaccine recipients and supported the resulting observations through equal cellular sampling, individual-participant analyses, expert gating, and functional confirmation of antigen specificity (Kramer et al., 2022). More recently, single-cell RNA sequencing of a typhoid CHIM cohort included six participants -three TD and three NoTD- and addressed inter-individual variability through participant-level analyses, local inverse Simpson’s index measurements, permutation testing, and false-discovery-rate correction (Bossel Ben-Moshe et al., 2025). In the present study, equal numbers of cellular events from each participant and outcome group were used to minimize cell-yield imbalance, clusters were initially identified without using clinical outcome labels, and the resulting populations were evaluated using complementary approaches, including PhenoGraph, T-REX, feature-ranking analysis, MEM, and conventional supervised gating. These measures provide internal support for the reproducibility and biological relevance of the identified patterns but do not eliminate participant-level overfitting or substitute for validation in an independent cohort. The absence of statistical significance between groups should not be interpreted as evidence of no biological difference but rather that more studies with larger cohorts are needed to confirm these CD8^+^ T findings. This may have precluded some of the trends (p ≤ 0.1) from reaching statistically significant values of differences in the various CD8^+^ T cells cluster levels, characteristics, and functions between TD and NoTD participants. Additionally, an important limitation of this study is that healthy adults enrolled in a non-endemic controlled human infection model may not fully represent individuals living in typhoid-endemic settings. Endemic populations may have pre-existing or repeatedly boosted immunity arising from prior exposure to *S.* Typhi, other *Salmonella* serovars, or antigenically related enteric organisms, in addition to differences in age, microbiota, nutrition, and environmental exposures. We have reported that the composition and functional characteristics of *S.* Typhi-responsive T_M_ is influenced by age (Rudolph et al., 2019a; Rudolph et al., 2019b; Booth et al., 2021). Taken together, these observations indicate that these factors may influence both the magnitude and quality of Ty21a-induced T cell immunity. Thus, the signature profiles identified in this healthy adult CHIM cohort should not be assumed that they extend directly to pediatric or older populations. As summarized by Levine in the “Typhoid Fever Vaccines” chapter of *Plotkin’s Vaccines*, Ty21a efficacy has varied across controlled challenge and field studies according to vaccine formulation, dosing regimen, age, intensity of exposure, duration of follow-up, and case definition (Levine, 2018). Three doses of enteric-coated Ty21a provided 67% protection over three years and 62% protection over seven years in a large field trial in Chile. Thus, the results herein should be interpreted cautiously because CHIM and field studies differ substantially in challenge dose, exposure frequency, diagnostic surveillance, case definition, follow-up duration, and background immunity. Nevertheless, the high-dose, closely monitored CHIM represents a stringent setting for identifying immune programs associated with clinical outcomes. The signatures identified here therefore require validation in endemic populations, where prior exposure and naturally acquired immunity may amplify, modify, or obscure the Ty21a-associated cellular programs observed in typhoid-naïve volunteers.

Finally, although our analyses identify strong associations between CD8^+^ T memory phenotypes and clinical outcomes, they do not establish causality. These caveats are consistent with the broader typhoid field, and human studies in general, where cellular correlates remain incompletely resolved despite substantial progress from challenge and vaccine studies (Fresnay et al., 2016). Furthermore, complementary analyses of the same CHIM cohort have also identified outcome-associated CD4^+^ cT_FH_ responses, including increased cT_FH_17 frequencies in NoTD participants, further supporting the contribution of coordinated CD4^+^ and CD8^+^ T-cell immunity to typhoid disease outcome.

In summary, our study supports a model in which protection from typhoid is associated with an early, multifunctional, phenotypically coordinated CD8^+^ T memory program that spans T_EM_, T_CM_, and T_EMRA_ subsets, whereas disease is associated with a distinct program marked by altered trafficking signatures and reduced early effector functionality. By integrating unsupervised clustering, T-REX analysis, feature ranking, and conventional analysis validation, we provide evidence that typhoid outcome is linked to the quality, timing, and subset distribution of *S*. Typhi-responsive CD8^+^ T memory responses. These findings refine current models of typhoid immunity and support the inclusion of high-dimensional CD8^+^ T memory profiling in future studies of oral vaccination and human challenges.

## Data Availability

The original contributions presented in the study are included in the article/**Supplementary Material**. Further inquiries can be directed to the corresponding authors.
