# Supplementary material for: Distinct time-dependent S. Typhi-specific CD8+ T cell programs are associated with clinical outcome following Ty21a immunization and wild-type S. Typhi challenge in humans
Source: Front Cell Infect Microbiol. 2026 Oct 2;16:1924754. doi: 10.3389/fcimb.2026.1924754 (PMC13671977; doi:10.3389/fcimb.2026.1924754)
Supplement: Supplementary file 1 [file DataSheet1.docx]

**Supplemental Figure Legends**

**Figure S1: Schematic of Typhoid** **Control Human Infection Model (CHIM).** Volunteers were recruited and immunized with three doses of the live oral attenuated typhoid vaccine, Ty21a, at days minus 28 (D-28), D-26 and D-24. The participants were then challenged on day 0 (D0) with wt *S*. Typhi (Quailes Strain) at a dose of 1-5 X 10^4^ CFU. At around day 7, some volunteers developed typhoid disease (TD) (blue) while others did not develop typhoid disease (NoTD) (red). On day 14, all volunteers (TD and NoTD) received antibiotics (Abx). PBMC were collected from multiple time points (shown in red) from baseline (D-28) up to 28 (D28) days after challenge. The number of participants studied for each time point in the TD and NoTD groups are shown.

**Figure S2: Workflow for computation of Gini importance scores used for outcome prediction.** Schematic overview of the feature engineering and random-forest modeling pipeline. PBMC samples stimulated with non-infected (NI) or *S*. Typhi-infected (ST) B-LCL targets were analyzed by CyTOF to quantify surface and intracellular marker expression and derive model features, including (i) frequency of manually gated T cell subsets and (ii) functional features defined by upper-quartile expression of phenotypic markers. A random-forest classifier was trained to predict clinical outcome (TD vs NoTD), and Gini importance was used to rank the contribution of each feature.

**Figure S3: Cluster marker patterns and cluster differences between TD vs NoTD and NI vs ST in CD8^+^T_EM_.** (**A**) Each cluster (1-17) expressed various markers in our panel at varying levels of expression as shown by color (red-maximum expression; blue-low/no expression). The frequency of each CD8^+^ T_EM_ cluster is compared between TD and NoTD participants by their color intensity as shown by the color yellow-black color scheme (Yellow-maximum frequency; black/dark blue-low/no frequency). (**B**) Furthermore, the frequency of each CD8^+^ T_EM_ cluster is compared between non-infected (NI) and *S*. Typhi-infected B-LCL (ST) from TD and NoTD using the yellow-black color scheme. Boxes show cluster with differences between groups.

**Figure S4: Determination of the 17 clusters in *S*. Typhi-specific CD8^+^ T_EM_ in NoTD and TD participants.** The net *S*. Typhi responses of each CD8^+^ T_EM_ clusters (1-17) were determined by stimulation of CD8^+^ T_EM_ with (i) *S*. Typhi-infected (ST) or (ii) non-infected (NI) autologous B-LCL. The net *S*. Typhi responses were calculated by the difference of ST to NI from participants in both TD and NoTD groups at all time points (D-28 to D28). Significant differences between TD (blue line and symbols) and NoTD (red line and symbols) participants at each time point are represented by * p<0.05. P-values are indicated for trends to show differences (0.05<p<0.1) between TD and NoTD groups for each *S*. Typhi-specific CD8^+^ T_EM_ clusters at each time point. Values are shown as median % of *S*. Typhi-specific events.

**Figure S5: Top immune CD8⁺ T cell clusters predicting NoTD and TD.** Outcome-predictive clusters are summarized across study phases (background, immunization, challenge), highlighting clusters associated with NoTD versus TD. Clusters are ranked from lowest to highest Gini importance score within each study phase and outcome group. Boxed regions highlight the four highest-ranking clusters in each comparison. Colored bars denote clusters with prominent outcome-associated importance scores. Cluster 5 (red bar) was prominent across all three phases in NoTD participants; Cluster 13 (orange) was prominent in both NoTD and TD participants at different study phases; Cluster 7 (green) was prominent during the challenge phase in both NoTD and TD participants; Cluster 2 (purple) showed the highest importance score and was most prominent in TD participants during background phase.

**Figure S6: Cluster marker patterns and cluster differences between TD vs NoTD and NI vs ST in CD8^+^ T_CM_.** (**A**) Each CD8^+^ T_CM_ cluster (1-17) expressed various markers in our panel at varying levels of expression as shown by color (red-maximum expression; blue-low/no expression). The frequencies of each cluster between TD and NoTD participants is compared by their color intensity shown by the color scheme (Yellow-maximum frequency; black/dark blue-low/no frequency). (**B**) In addition, the frequency of each CD8^+^ T_CM_ cluster between non-infected (NI) and *S*. Typhi-infected B-LCL (ST) is compared by Yellow-black color scheme.

**Figure S7: Determination of *S*. Typhi-specific CD8^+^ T_CM_ clusters in NoTD and TD participants.** The net *S*. Typhi responses of each CD8^+^ T_CM_ clusters (1-17) were determined by stimulation of CD8^+^ T_CM_ with (i) *S*. Typhi-infected (ST) or (ii) non-infected (NI) autologous B-LCL. The net *S*. Typhi responses were calculated by the difference of ST to NI from participants in both TD and NoTD groups at all time points (D-28 to D28). Values are shown as median % of *S*. Typhi-specific events.

**Figure S8: Cluster marker patterns and cluster differences between TD vs NoTD and NI vs ST in CD8^+^ T_EMRA_.** (**A**) Each CD8^+^ T_EMRA_ cluster (1-17) expressed various markers in our panel at varying levels of expression as shown by color (red-maximum expression; blue-low/no expression). The frequencies of each cluster between TD and NoTD participants is compared by their color intensity shown by the color scheme (Yellow-maximum frequency; black/dark blue-low/no frequency). (**B**) In addition, the frequency of each CD8^+^ T_EMRA_ cluster between non-infected (NI) and *S*. Typhi-infected B-LCL (ST) is compared by yellow-black color scheme.

**Figure S9: Determination of *S*. Typhi-specific CD8^+^ T_EMRA_ clusters in NoTD and TD participants.** The net *S*. Typhi responses of each CD8^+^ T_EMRA_ clusters (1-17) were determined by stimulation of CD8^+^ T_EMRA_ with (i) *S*. Typhi-infected (ST) or (ii) non-infected (NI) autologous B-LCL. The net *S*. Typhi responses were calculated by the difference of ST to NI from participants in both TD and NoTD groups at all time points (D-28 to D28). Values are shown as median % of *S*. Typhi-specific events.

**Figure S10: ROC statistics for discrimination between NoTD- and TD-associated CD8^+^ T_CM_ and T_EMRA_ markers.** ROC curves were used to assess the statistical differences between TD and NoTD marker expression as determined by conventional supervised gating analysis which are shown in the graphs of **Fig. 13 A-I**. AUC and *p* values are indicated within each panel.
